# Identifying gene variants underlying the pathogenesis of diabetic retinopathy based on integrated genomic and transcriptomic analysis of clinical extreme phenotypes

**DOI:** 10.3389/fgene.2022.929049

**Published:** 2022-08-10

**Authors:** Qiaoling Song, Yuchao Zhang, Minghui Zhang, Xiaoli Ma, Qianyue Zhang, Chenyang Zhao, Zhongwen Zhang, Huichen Zhao, Wenchao Hu, Xinxin Zhang, Xiwen Ren, Ming An, Jinbo Yang, Yuantao Liu

**Affiliations:** ^1^ School of Medicine and Pharmacy, Ocean University of China, Qingdao, China; ^2^ Innovation Platform of Marine Drug Screening and Evaluation, Qingdao National Laboratory for Marine Science and Technology, Qingdao, China; ^3^ Department of Endocrinology, Qingdao Municipal Hospital, Qingdao, China; ^4^ Department of Endocrinology and Metabolism, the First Affiliated Hospital of Shandong First Medical University and Shandong Provincial Qianfoshan Hospital, Jinan, China; ^5^ Department of Endocrinology, Qilu Hospital (Qingdao), Cheeloo College of Medicine, Shandong University, Qingdao, China; ^6^ Department of Emergency, Linyi People’s Hospital, Linyi, China; ^7^ Department of Ophthalmology, Qingdao Municipal Hospital, Qingdao, China

**Keywords:** diabetic retinopathy, exome sequencing, rare variants, pathogenic mutation, RNA-seq analysis

## Abstract

Diabetic retinopathy (DR) is a common complication and the leading cause of blindness in patients with type 2 diabetes. DR has been shown to be closely correlated with blood glucose levels and the duration of diabetes. However, the onset and progression of DR also display clinical heterogeneity. We applied whole-exome sequencing and RNA-seq approaches to study the gene mutation and transcription profiles in three groups of diabetic patients with extreme clinical phenotypes in DR onset, timing, and disease progression, aiming to identify genetic variants that may play roles in the pathogenesis of DR. We identified 23 putatively pathogenic genes, and ingenuity pathway analysis of these mutated genes reveals their functional association with glucose metabolism, diabetic complications, neural system activity, and dysregulated immune responses. In addition, ten potentially protective genes were also proposed. These findings shed light on the mechanisms underlying the pathogenesis of DR and may provide potential targets for developing new strategies to combat DR.

## Introduction

Diabetic retinopathy (DR) is a common microvascular complication of diabetes alongside polyneuropathy and nephropathy (Cheung et al., 2010), and it is also the main cause of vision loss among working-age people (Cheung et al., 2010; Cheung et al., 2014; Lin et al., 2014; Gella et al., 2017). More than 60% of patients with type 2 diabetes (T2DM) will develop some degree of DR within 20 years after diagnosis (Fong et al., 2004; Jenkins et al., 2015; Wong et al., 2016), and 1.6% of elder-onset T2DM patients are legally blind (Fong et al., 2004). The estimated number of people with DR will be 191.0 million by 2030 (Zheng et al., 2012).

The pathological characteristics of DR include leaky blood vessels, formation of microaneurysms, presence of protein exudates in the vitreous body, and retinal neovascularization, which eventually lead to a steady decline in visual acuity and even vision-threatening complications, such as vitreous hemorrhage and retinal detachment (Alghadyan, 2011; Whitehead et al., 2018).

Numerous clinical studies show that the risk of DR development is closely correlated with blood glucose levels and the duration of diabetes (Chaudhury et al., 2017). However, other studies show that levels of hemoglobin A1c (HbA1c) and disease duration only account for 11% of the retinopathy risk (Hirsch and Brownlee, 2010) and individuals with very well-controlled blood sugar levels may or may not develop DR. Moreover, DR usually has slow progression over decades after the initial diagnosis of diabetes, from mild to moderate to severe nonproliferative retinopathy (NPDR) and finally most advanced proliferative retinopathy (PDR) (Alghadyan, 2011). However, it was noted in clinic that some newly diagnosed or new-onset diabetic patients may develop NPDR or even PDR in a very short period (Stratton et al., 2001). Although hyperglycemia, hypertension, and dyslipidemia were reported to be the risk factors (Ebneter and Zinkernagel, 2016; ValdezGuerrero et al., 2021), accumulating evidence suggests that DR, especially PDR, could be a heritable condition with an estimated heritability of 27–50% (Arar et al., 2008; Hietala et al., 2008). Several candidate genes associated with the pathogenesis of DR have been proposed, including *AKR1B1* (Kaur and Vanita, 2016), *GRB2*, *NOX4*, and *NVL* (Cole and Florez, 2020). By contrast, *NME3*, *LOC728699*, and *FASTK* were proposed to have protective effects against DR (Shtir et al., 2016). These findings suggested that genetic variants of either the pathogenic or protective genes may play important roles in the development of DR independent of glucose control. In addition, inflammation is also suggested to play a role in the development of DR (Xu and Chen, 2017). However, to what extent the above factors may contribute to the pathogenesis of DR remains largely unknown.

In the present study, three extreme phenotypes of T2DM patients within the Han population were studied. The early-onset (DR) group includes patients who developed DR within a median time of 1 year after the onset of T2DM; the non-DR group (DM) includes patients who had no DR at least 10 years after the onset of T2DM; the late-onset DR group (DM–DR) includes patients who had the first diagnosis of DR at least 10 years after the onset of T2DM. By applying the whole-exome sequencing (WES), we identified the putative pathogenic genes associated with early-onset DR and further verified them in the RNA-seq data of the same sample in this study as well as with the previously published data from other groups. The ingenuity pathway analysis (IPA) of these mutated genes reveals that their functions link with not only the well-known glucose metabolism, diabetic complications, but also neural system activity and dysregulated immune responses, indicating that DR is a complex disease, and its progression might be influenced by multiple genes regarding multiple pathophysiological aspects. Moreover, we identified some potential protective genes by comparing the differentially mutated genes (DMGs) in the DM group with DR or DM–DR groups.

## Materials and methods

### Participants

A total of 15 subjects covering three extreme phenotypes of T2DM were recruited for this study. All subjects underwent a physical examination. Body weight and height were measured in a standardized procedure, and body mass index (BMI) was defined as weight in kilograms divided by the square of height in meters (kg/m^2^). Blood pressure was measured twice with a Mercury sphygmomanometer at 3-min intervals from the right arm in the sitting position after 5 min rest, and the mean value was calculated.

The levels of blood glucose and C-peptide were measured using an enzymatic method and a radioimmunoassay kit (Roche Diagnostics, Germany), respectively. Serum HbA1c was measured using high-pressure liquid chromatography, and serum triglyceride and total cholesterol, high-density lipoprotein cholesterol, and free fatty acid levels were measured using an autoanalyzer (Modular E170, Roche). Antiglutamic acid decarboxylase antibody (GADA) was measured via chemiluminescent immunoassay (YHLO, iFlash 3000-A).

Diabetes was diagnosed according to the American Diabetes Association (2020). Duration of diabetes was calculated from the time of the first occurrence of hyperglycemia rather than the time of diagnosis. Patients were excluded if they fulfilled the criteria of T1D: positive for GADA or fasting C-peptide less than 0.8 ng/ml at the onset of diabetes. The diagnosis of DR and DR staging was made based on ophthalmoscopy and fluorescein angiography by experienced ophthalmologists and referred to the “Proposed International Clinical Diabetic Retinopathy and Diabetic Macular Edema Disease Severity Scales” by the Global Diabetic Retinopathy Project Group (Wilkinson et al., 2003). In a word, DR staging criteria are as follows: stage 0: no apparent retinopathy; stage I: mild nonproliferative DR, microaneurysms only; stage II: moderate nonproliferative DR, more than just microaneurysms but less than severe nonproliferative DR; stage III: severe nonproliferative DR, any of the following: more than 20 intraretinal hemorrhages in each of four quadrants; definite venous beading in 2 + quadrants; prominent intraretinal microvascular abnormalities in 1 + quadrant and no signs of proliferative retinopathy; stage IV: proliferative DR, one or more of the following: neovascularization, vitreous/preretinal hemorrhage. The early-onset (DR) group included five patients who developed DR within a median time of 1 year after the onset of T2DM; the non-DR group (DM) included six patients who had no DR at least 10 years after the onset of T2DM; the late-onset DR group (DM–DR) included four patients who had the first diagnosis of DR at least 10 years after the onset of T2DM. For a comprehensive description of phenotypic and diabetic clinical classification see Table 1. This study was approved by the Ethics Committee of Qingdao Municipal Hospital, and all patients provided written informed consent.

**TABLE 1:** Clinical data summary

Variable	DM	DR	DM–DR
	*n* = 6	*n* = 5	*n* = 4
Age, years	62.83 ± 9.52	45.6 ± 7.3	74.75 ± 3.5
Gender M	2	1	2
F	4	4	2
Diagnosis	T2DM	T2DM	T2DM
Medical history, years	20.5	3.5	24
Range	10–22	1–5	10–38
Retinopathy	0 (6)	5 (5)	4 (4)
Stage	N	II–IV	II–IV
Macroangiopathy	Y6N0	Y3N2	Y4N0
Microangiopathy (nephropathy)	Y1N5	Y4N1	Y2N2
Peripheral neuropathy	Y1N5	Y1N4	Y4N0
BMI (kg/m^2^)	23.6 ± 2.94	23.6 ± 1.72	24.3 ± 2.95
Hypertension	3 (6)	4 (5)	2 (4)
SBP (mmHg)	145.83 ± 20.39	147.8 ± 6.57	136.75 ± 2.95
DBP (mmHg)	92.5 ± 8.8	91.4 ± 5.5	79 ± 15.64
HbA1c % (mmol/mol)	8.85 ± 1.66	11.36 ± 3.58	9.13 ± 1.11
C-peptide	2.34 ± 0.56	2.05 ± 0.44	1.56 ± 0.35
TC (mmol/L)	4.83 ± 1.58	6.14 ± 0.68	4.07 ± 0.82
TG (mmol/L)	3.95 ± 4.55	2.74 ± 2.33	2.39 ± 1.11
FFA (mmol/L)	0.61 ± 0.31	0.48 ± 0.21	0.46 ± 0.19
HDL-c (mmol/L)	1.18 ± 0.46	1.22 ± 0.24	0.97 ± 0.27
LDL-c (mmol/L)	2.5 ± 0.77	3.61 ± 0.61	2.22 ± 0.4

Note: Data are expressed as the mean ± SD. M, male; F, female; BMI, body mass index; SBP, systolic blood pressure; DBP, diastolic blood pressure; HbA1c, hemoglobin A1c; TG, triglyceride; TC, total cholesterol; FFA, free fatty acid; HDL-c, high-density lipoprotein cholesterol; and LDL-c, low-density lipoprotein cholesterol.

### Whole-exome sequencing

The plasma and peripheral blood mononuclear cells (PBMCs) were separated via Ficoll Density gradient centrifugation (RT, 300 g, 10 min). The DNA and RNA of PBMCs were isolated and processed for WES and RNA-seq, respectively.

The SureSelect XT HS target enrichment workflow system was used to capture exonic fragments for Illumina Multiplexed Sequencing as described in the protocol provided by Agilent Technologies. For WES, 3 μg genomic DNA from each sample was sheared into fragments of 150–200 bp. Enriched exome libraries were multiplexed and sequenced on the HiSeq 2,500 platform (Illumina, United States). A paired-end DNA sequencing library was prepared via gDNA shearing, end-repair, A-tailing, paired-end adaptor ligation, and amplification. After hybridizing the library with bait sequences for 16 h, the captured library was purified and amplified with an indexing barcode tag, and library quality and quantity were assessed using a 2,200 TapeStation Instrument and Qubit 2.0 Fluorometer, respectively. The exome library was sequenced using the 10 bp paired-end mode of the TruSeq Rapid PE Cluster kit and the TruSeq Rapid SBS kit (Illumina).

### Data analysis of whole-exome sequencing

To distinguish the possible rare variants from these numerous mutation changes, ANNOVAR (Wang et al., 2010) was employed to annotate the single-nucleotide polymorphism (SNP) and Indel of the provided genome through databases and comply functional prediction of variant loci through tools, including deleterious prediction (SIFT value ≤ 0.05), low-frequency calculation (1,000 Genomes/2015aug_all, esp6500siv2_all and ExAC_EAS < 0.05), and pathogenic/likely pathogenic annotation by Clinvar. Hierarchical clustering of all the patients was applied to study the mutation pattern within each group. In addition, the Pearson correlation was drawn to describe the correlation among the DMGs for these three groups (*p*-value < 0.05 and Pearson correlation score ≥ 0.8). The top-ranked DMGs were imported into IPA to visualize their relationship with diabetic complication-associated networks.

### Transcriptome sequencing (RNA-seq)

Total RNA was isolated using the Trizol Reagent (Invitrogen Life Technologies), after which the concentration, quality, and integrity were determined using a NanoDrop spectrophotometer (Thermo Scientific). Of the RNA, 3 μg was used as input material for the RNA sample preparations. Sequencing libraries were generated using the TruSeq RNA Sample Preparation Kit (Illumina, San Diego, CA, United States). To select cDNA fragments of the preferred 200 bp in length, the library fragments were purified using the AMPure XP system (Beckman Coulter, Beverly, CA, United States). DNA fragments with ligated adaptor molecules on both ends were selectively enriched using Illumina PCR Primer Cocktail in a 15-cycle PCR reaction. Products were purified (AMPure XP system) and quantified using the Agilent high sensitivity DNA assay on a Bioanalyzer 2,100 system (Agilent). The sequencing library was then sequenced on a HiSeq platform (Illumina).

### Data analysis of RNA-seq

After passing the quality inspection, raw data (raw reads) were processed using Trimmomatic. The reads containing ploy-N and the low-quality reads were removed to obtain clean reads using Cutadapt (v1.15) software. Then, the clean reads were mapped to the reference genome using HISAT2 (Kim et al., 2015) (http://ccb.jhu.edu/software/hisat2/index.shtml), and the default mismatch was no more than 2. The alignment region distribution of mapped reads was calculated. The FPKM value of each gene was calculated using cufflinks (Roberts et al., 2011), and the read counts of each gene were obtained using htseq-count (Anders et al., 2015). Differentially expressed genes (DEGs) were identified using the DESeq. *p*-value < 0.05 and fold change > 2 or fold change < 0.5 were set as the threshold for significantly differential expression. Hierarchical cluster analysis of DEGs was performed to explore gene expression patterns. Gene set enrichment analysis (GSEA) (Subramanian et al., 2005), IPA (Krämer et al., 2014), and Metascape (Zhou et al., 2019) analysis of DEGs were performed. The immune cell abundance identifier (ImmuCellAI) and Timer2 platforms were applied for the evaluation of the immune cell abundance from the RNA-seq data.

### Validate identified variations with public RNA-seq datasets of eye tissues

To verify the DMGs in our exome sequencing, data from the RNA-seq public database of eye tissues with DR (GSE102485 and GSE94019) were processed for mutation analysis. Canonical pathway analysis by IPA was performed to identify the functions of the shared mutations.

### Cytometric bead array system

The plasma immunoglobulin subtypes produced by B cells were measured using cytometric bead array analysis (BD Biosciences, Cat.550026), including IgG1, IgG2, IgG3, IgG4, IgA, IgD, IgE, and IgM.

## Results

### Putative pathogenic genes were identified for diabetes and diabetic retinopathy

**FIGURE 1:**
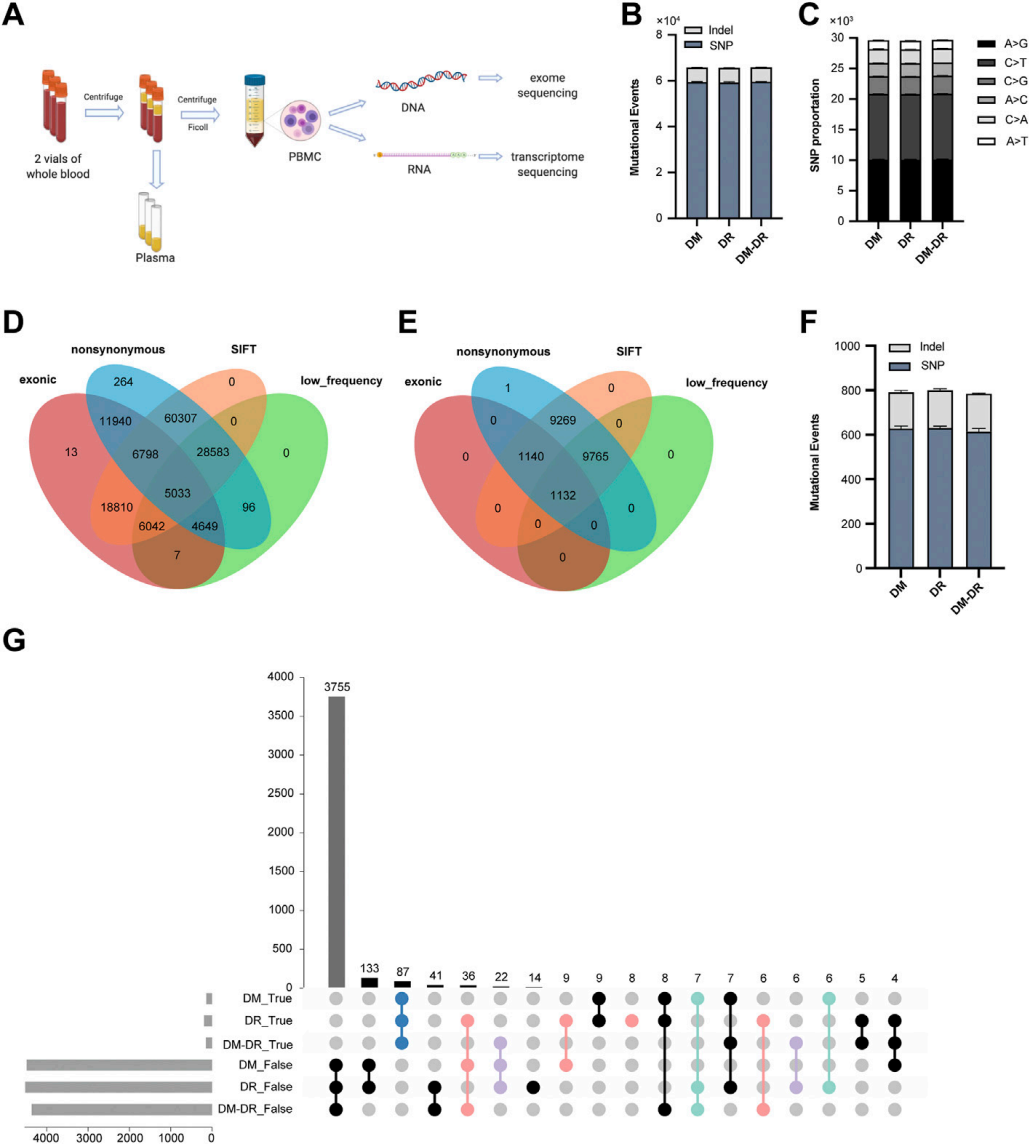
Exome-based pathogenic evaluation of patient peripheral blood mononuclear cells (PBMCs). **(A)** schematic diagram of sample processing. Peripheral blood samples were collected and plasma and PBMCs were separated. The DNA and RNA were isolated from PBMCs and processed for high-throughput RNA and DNA sequencing, respectively. **(B)** histogram of the numbers of mutational events in each group. **(C)** base-level transitions and transversions in each group shown in **(B)**. **(D,E)** Venn diagram for the comparison of number of single-nucleotide polymorphisms **(D)** and Indels **(E)** called between different mutation calling tools using the ANNOVAR software. **(F)** histogram of the numbers of pathogenic mutational events in each group. **(G)** UpSet plot showing the intersection of overlapped pathogenic variants across three groups. True represents mutation rate ≥ 60% and false represents mutation rate ≤ 40%.

The sample processing workflow for RNA and exome sequencing were shown in Figure 1A. The clinical characteristics of patients and their diagnosis criteria for each group were summarized in Table 1. All the patients diagnosed with T2DM have comparable BMI values. Every patient in DM and DM–DR groups and 3 of 5 DR patients were affected with macroangiopathy and 4 of 5 DR patients were with microangiopathy (nephropathy). Exome capture, sequencing, and data processing of all the samples identified a total of 168,544 mutations and 56,046 were protein-altering (15,858 missense, 11,808 nonsynonymous single-nucleotide variants (variation in a single nucleotide without any limitations of frequency), 2,522 insertion/deletion and 508 stop loss/gain). There are no obvious differences in mutational events among these three groups (Figure 1B) as well as the base-level mutation spectrum in which A-to-G and C-to-T transversions were the most common changes (Figure 1C). To distinguish the possible rare variants from these numerous mutation changes, ANNOVAR (Wang et al., 2010) was employed to annotate the SNP (a substitution of a single nucleotide that occurs at a specific position in the genome, where each variation is present to some appreciable degree within a population) and Indel of the provided genome through databases, such as 1,000 Genomes/2015aug_all, esp6500siv2_all, ExAC_EAS, and SIFT values, and comply functional prediction of variant loci through tools, such as Clinvar annotation. In total, we identified 5,033 SNP and 1,132 Indel candidate variants after filtering nonsynonymous exonic regions by deleterious prediction (SIFT value ≤ 0.05) and low-frequency calculation (1,000 Genomes/2015aug_all, esp6500siv2_all and ExAC_EAS < 0.05) or pathogenic/likely pathogenic annotation by Clinvar (Figures 1D,E). The pathogenic events of each group were calculated, and no significant differences in gene numbers were observed (Figure 1F). Since multiple mutational variants were frequently identified in one gene, for example, *CTBP2* with three variant sites in chr10: 126686629, 126683071, and 126683123 which are all predicted as nonsynonymous and pathogenic candidates, pathogenic genes instead of pathogenic variants were summarized and mutation pattern of these three groups were analyzed (Figure 1G). There are 87 genes shared in all the groups putatively related to diabetes occurrence (mutation rate ≥ 60% in each group was defined as true mutation). Pathogenic mutant genes respectively enriched in the indicated groups might participate in the pathogenesis of DR (red, susceptible mutant genes), DM–DR (purple, susceptible mutant genes), and DM (green, protective mutant genes). Overall, numerous pathogenic mutational variants and genes were identified through high-throughput exome sequencing and putatively different among these three groups that have distinct clinical phenotypes.

### Candidate genes prioritized for the pathogenesis of retinopathy

**FIGURE 2:**
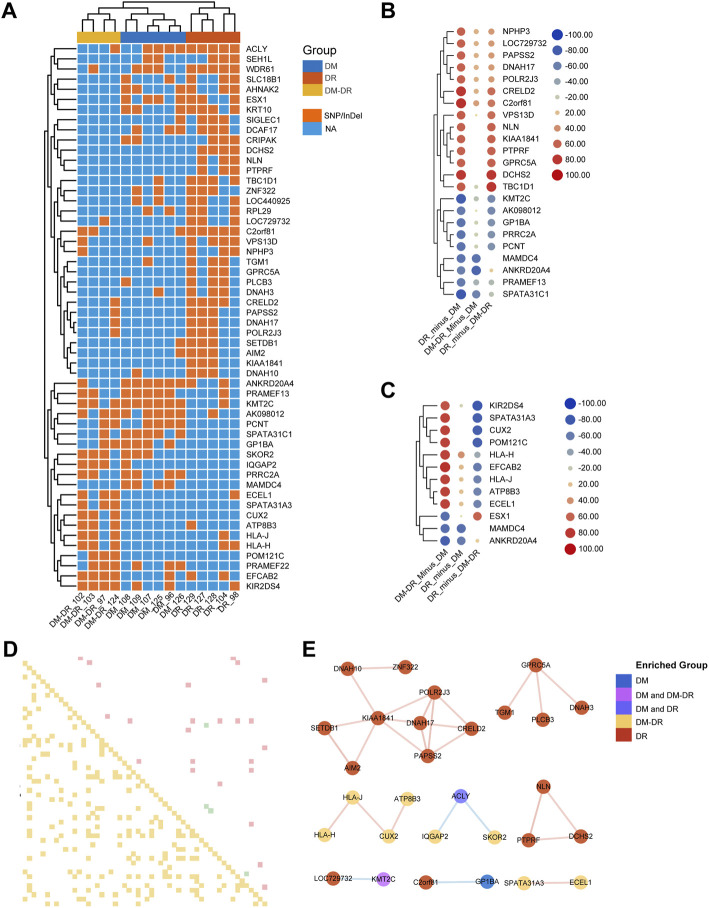
Putatively susceptible or protective mutant genes detected across the groups. **(A)** Heat map-based hierarchical clustering of the 54 putatively pathogenic genes separates these three groups. Under each cluster, samples were sub-grouped by their patients of origin. **(B)** the 23 significant differentially mutant genes (DMGs) between diabetic retinopathy (DR) and non-DR (DM) groups. **(C)** the 12 significant DMGs between DM–DR and DM groups. The mutation significance cutoff is 60%. **(D)** Pearson correlation chart of sigDMGs across 15 samples. The upper off-diagonal entry denotes the pairwise correlation level (red box represents correlation score ≥ 0.8, green ≤ −0.8) and lower off-diagonal denotes the corresponding *p*-values (yellow box represents *p*-value < 0.05). **(E)** correlation network based on Pearson correlation coefficients among these three groups. The red lines indicate positive correlation, and the blue ones indicate negative correlation.

Through stringent filtering (mutation rate difference ≥ 60% among comparison groups), a total of 54 genes were screened out to represent the significant difference (Figure 2A). The representative SNP and Indel variants were listed in Supplementary Table S1. Hierarchical clustering of all the patients indicated that mutation pattern within each group was respectively consistent, and the DR group was relatively distant from DM and DM–DR with more different mutant genes. Although patients in both DR and DM–DR groups are accompanied by severe retinopathy, only part of the significantly different DMGs of the DR group compared with the DM group exhibits similar patterns to DMGs of the DM–DR group compared with the DM group (Figure 2B). Approximately half of these DMGs were specifically enriched or excluded in the DR group compared with both DM and DM–DR groups (Figure 2B), and so did the significant DMGs of DM–DR compared with DM (Figure 2C). These data suggested that the early-onset (DR) and late-onset (DM–DR) DR have both similarities and differences in mutation preferences when compared with no-retinopathy diabetes (DM). In addition, we draw the Pearson correlation among the 54 DMGs for these three groups (Figure 2D, *p*-value < 0.05 and Pearson correlation score ≥ 0.8) and a few general trends seem to emerge (Figure 2E). Among them, 16 genes (red) with rare mutations mainly in the DR group were positively correlated and grouped into three clusters, whereas six mutant genes enriched in DM–DR (yellow) were positively correlated and grouped into two clusters. There were also seven mutant genes enriched in one or two groups that were negatively correlated and grouped in three subsets. Besides, the possible relationship of DMGs with clinical characters was analyzed and different correlation patterns existed for different DMGs, manifested as different hierarchical clustering results (Supplementary Figure S1). These data suggested that a panel of mutant genes might simultaneously or exclusively account for the pathogenic retinopathy in T2DM.

Besides the generally used WES technique for exome mutation calling, RNA-seq data could also be utilized to identify genomic variants (Piskol et al., 2013). A total of 58,466 SNPs and 347 Indels in exome sequencing were verified by the variants identified with RNA-seq data (Supplementary Figures S2A,B). Approximately one-third of SNPs in each sample of RNA-seq were overlapped with exome sequencing results (Supplementary Figure S2C), and less indel mutations were identified in RNA-seq data potentially due to the technique limitation (Sun et al., 2017) (Supplementary Figure S2D). Among them, 20 out of 54 pathogenic DMGs were verified and relatively conserved within each group (Supplementary Figure S2E), confirming the significant differences among these groups.

### Function and disease annotation of the top-ranked mutant genes

**FIGURE 3:**
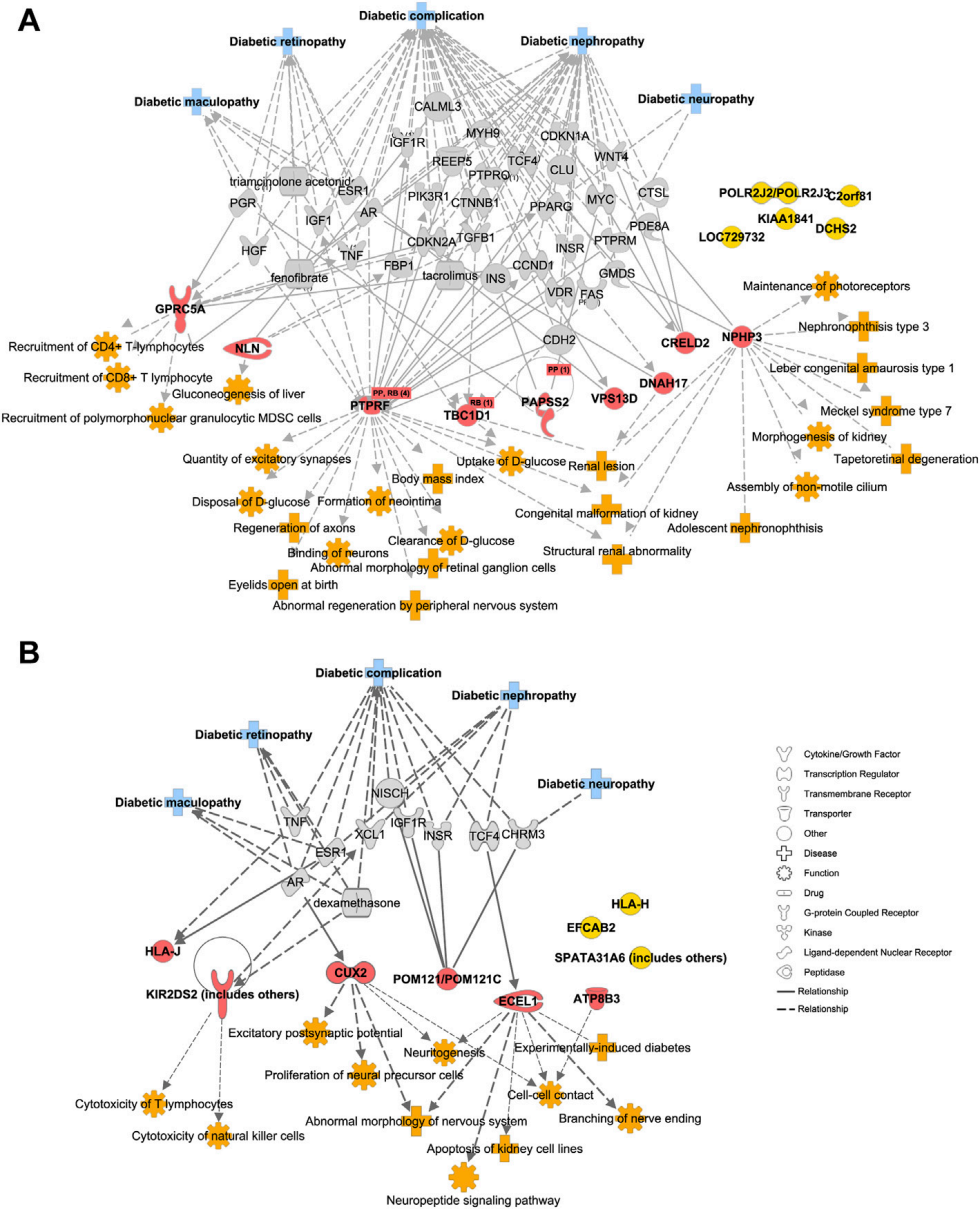
Diabetic complication-related disease and function analysis of putatively pathogenic mutant genes enriched in patients with oculopathy. **(A,B)** the network of diseases and functions by ingenuity pathway analysis highlights the relationships between diabetic complications and the putatively susceptible DMGs of DR vs. DM **(A)** and DM–DR vs. DM **(B)**, and functional enrichment of these genes, respectively.

We further imported 14 top-ranked mutant genes of DR vs. DM into IPA (Krämer et al., 2014) to visualize their relationship with diabetic complication-associated networks, including diabetic complication (1,177 genes), diabetic maculopathy (48 genes), DR (300 genes), diabetic neuropathy (97 genes) and diabetic nephropathy (879 genes) (Figure 3A, Supplementary Table S2). Nine of fourteen genes were predicted to be directly or indirectly connected with these complications, whereas the other five genes enriched in DR patients were not yet reported in previously published literatures. Since DR is usually accompanied by other diabetic complications, such as nephropathy and neuropathy (Cole and Florez, 2020), the mutant genes, such as *PTPRF* and *CRELD2* were linked with more than one type of complication, including DR. The IPA also revealed a direct connection with eye diseases and functions, such as maintenance of photoreceptors, abnormal morphology of retinal ganglion cells, and tapetoretinal degeneration. Some indirect diseases and functions were also enriched, which included immune cells especially T cell activation, glucose metabolism, renal abnormality, and nerve dysfunction. These results suggested that early-onset DR might result from common upstream pathophysiologic mechanisms also underlying disabilities of the retina, kidney, immune system, nerve tissues, and the regulation of global blood glucose. Likewise, the top-ranked mutant genes in the DM–DR group compared with the DM group were interconnected with various diabetic complications as in Figure 3A via IPA pathway explorer analysis (Figure 3B, Supplementary Table S3). Among them, three of nine genes were linked to DR or maculopathy whereas two genes were linked to diabetic nephropathy or neuropathy. Different from early-onset DR patients, disease and function analysis of late-onset DM–DR patients highlighted the immune cell activation and nerve dysfunction, and to less extent kidney disease. There were no functions of abnormal glucose metabolism enriched in the DM–DR group potentially because of the relatively better glycemic control before retinopathy occurrence in these long-term diabetic patients. Besides the above-mentioned susceptible mutant genes, we also identified a panel of putatively protective mutant genes in DM patients (Figures 2A–C). Two genes *MAMDC4* and *ANKRD20A4* overlapped in protective DMGs in DR vs. DM and DM–DR vs. DM groups. One gene *ESX1* was specifically enriched in DM–DR vs. DM, whereas seven genes were specifically enriched in DR vs. DM. All these 10 genes were evaluated for function and disease prediction by IPA and related to diabetic complications, including DR (Supplementary Figure S3, Supplementary Table S4). Development-associated functions and platelet behavior were involved in gene mutation-induced pathogenic changes in DM patients. These findings suggested that the putatively susceptible and protective mutant genes might participate in the functional regulation of local eye tissues, as well as the immune and metabolism systems to cause or prevent retinopathy.

### Correlation with public RNA-seq-based mutation mapping of pathogenic ocular tissues

**FIGURE 4:**
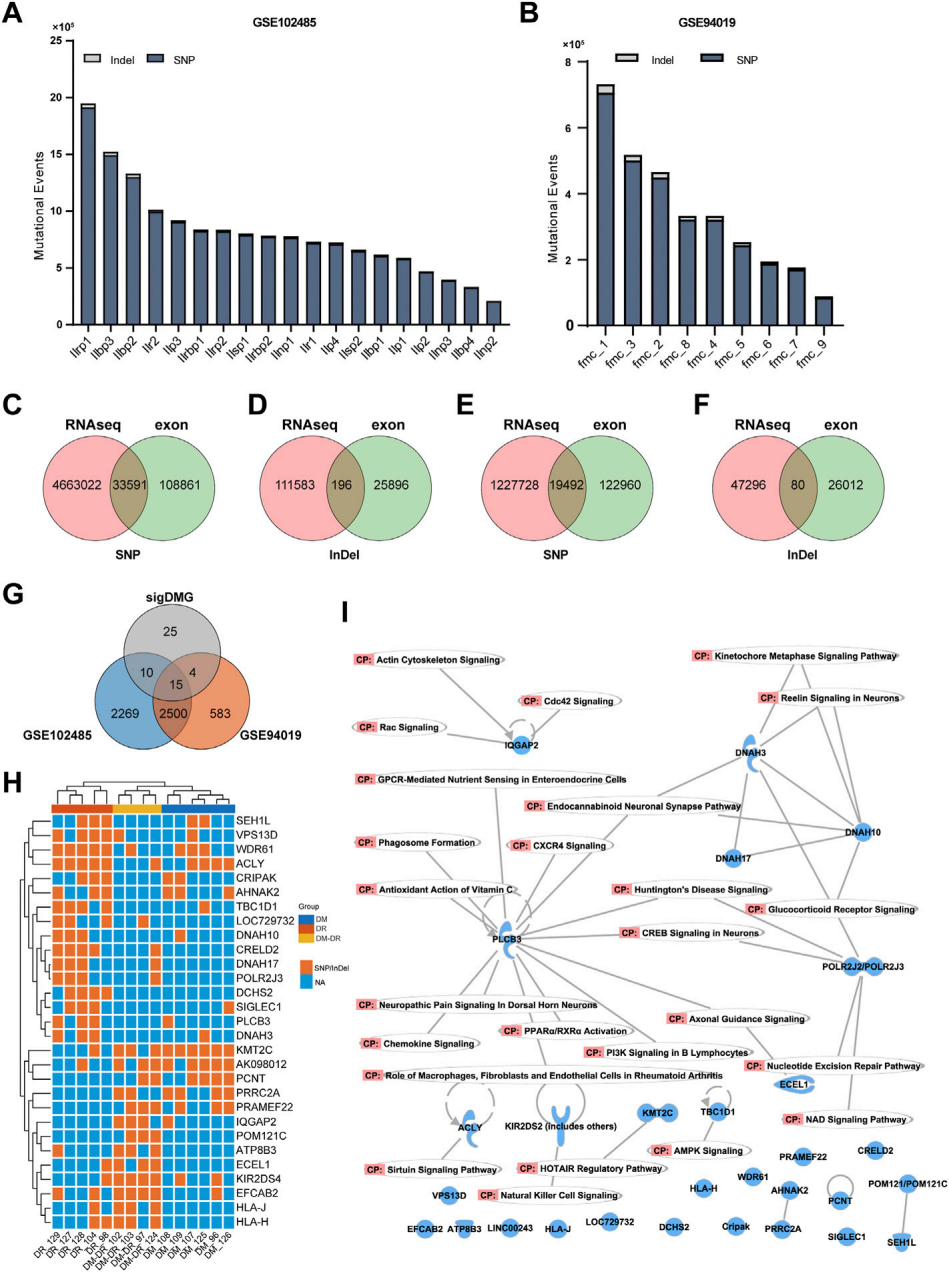
Mutant genes verified using mutation data of eye tissues of patients with diabetic retinopathy (DR). **(A,B)** overview of the numbers of mutational events from the DR-related RNA-seq datasets of GSE102485 **(A)** and GSE94019 **(B)**. **(C–F)** single-nucleotide polymorphism and Indel variants of exome sequencing were validated by RNA-seq data of GSE102485 **(C,D)** and GSE94019 **(E,F)**. **(G)** Venn diagram for the comparison of the 54 significant DMGs and exonic nonsynonymous mutational genes from RNA-seq datasets. **(H)** heat map-based hierarchical clustering of the overlapped 29 putatively pathogenic genes in **(G)** separates these three groups. Under each cluster, the samples were sub-grouped by their patients of origin. **(I)** canonical pathway analysis by ingenuity pathway analysis of the overlapped 29 genes.

Besides the generally used WES technique for exome mutation calling, RNA-seq data could also be utilized to identify genomic variants (Piskol et al., 2013). Due to the popularity and low cost of the RNA-seq technique for gene expression profiling, numerous RNA-seq data were published and shared through the public database, including eye tissues with DR (GSE102485 and GSE94019). GSE102485 represents 30 transcriptome profiles of neovascular proliferative membrane specimens, including 19 type II DR samples (Li et al., 2019); GSE94019 represents 13 RNA-seq datasets, including nine proliferative DR fibrovascular membrane samples (Lam et al., 2017). Therefore, we collected RNA-seq data from those 19 samples from GSE102485 and nine samples from GSE94019 with DR, which were processed for mutation analysis. A large number of mutational events were identified (Figures 4A,B), of which 33,787 mutational events in GSE102485 and 19,572 in GSE94019 were included in the genomic variances identified in our exome sequencing (Figures 4C–F). Furthermore, we compared our significant DMGs with the shared nonsynonymous exonic mutations detected in these samples, and 29 out of 54 DMGs were confirmed (Figure 4G). These 29 DMGs were mainly enriched in both DR and DM–DR groups and displayed high consistency within each group (Figure 4H). Using canonical pathway analysis by IPA, these DMGs were functionally enriched in the immune cell functions and nervous signaling pathways, such as CXCR4 signaling, natural killer cell signaling, axonal guidance signaling, and endocannabinoid neuronal synapse pathway (Figure 4I), highlighting the essential roles of the immune system and neural network in DR.

Chromosomal rearrangements juxtapose different genes together to form rare fusion genes and more severe genomic changes (Mertens et al., 2015). The genes involved in fusion genes were compared across all the samples from three groups and no more than two genes were shared in patients (Supplementary Figure S4A). Although no fusion gene patterns were noticed in Supplementary Figure S4A, the fusion gene number was higher in the late-onset retinopathy group DM–DR compared with the DM and DR groups (Supplementary Figure S4B), suggesting the possible contribution of gene fusion to pathogenic ocular disease at the late stage of diabetes.

### Transcriptome profiling immune status associated with diabetic retinopathy

**FIGURE 5:**
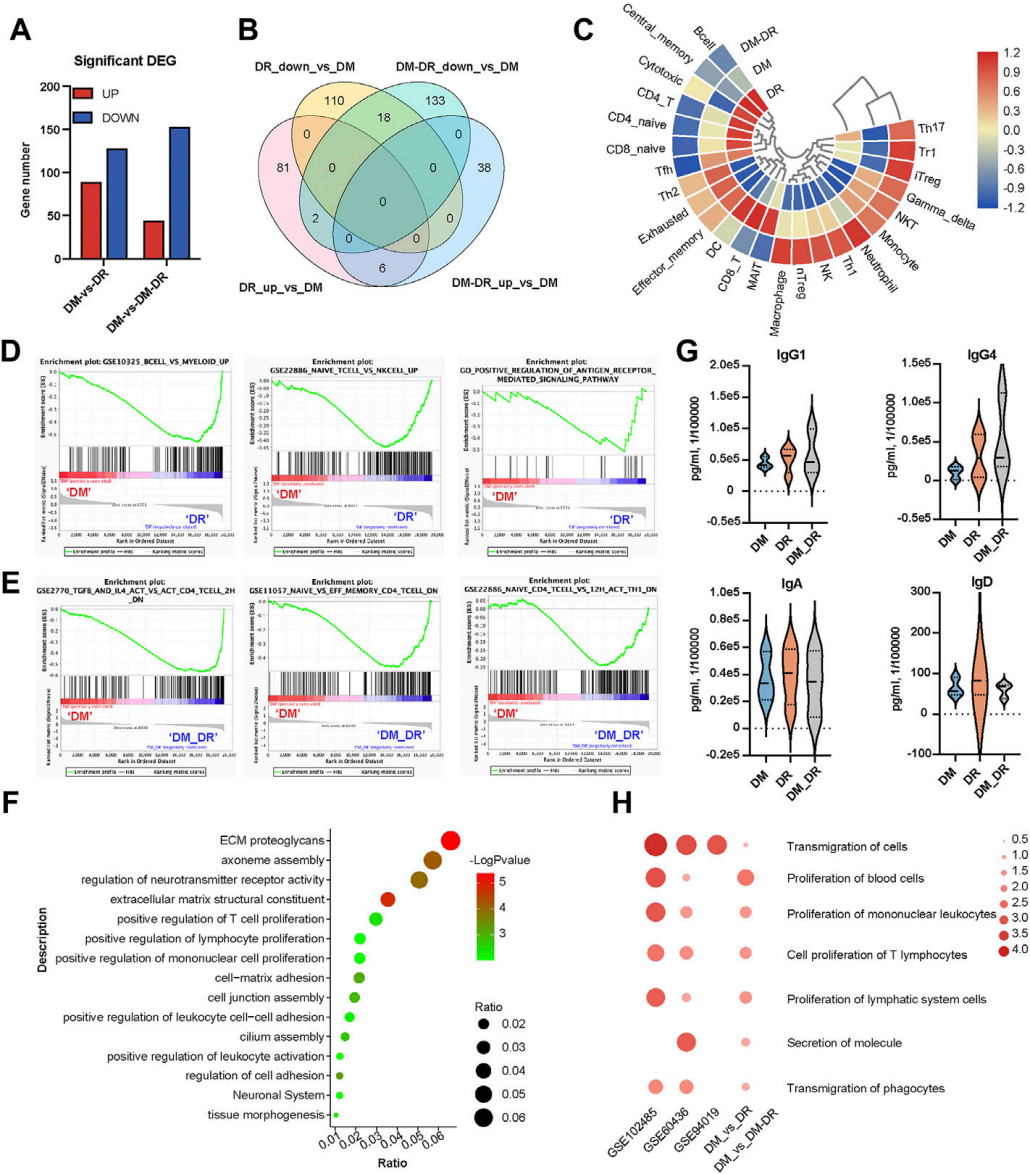
Diabetes-related disease and function analysis of differently expressed genes (DEGs) enriched in patients with oculopathy. **(A)** bar chart of DEGs among diabetic retinopathy (DR), non-DR (DM), and DM–DR groups. **(B)** Venn diagram of DEGs among the indicated comparison groups. **(C)** heat map-based ImmuCellAI and Timer2 platforms of the immune cell abundance from the RNA-seq data. **(D,E)** gene set enrichment analysis datasets enriched in DR **(D)** and DM–DR **(E)** upregulated gene clusters compared with the DM group. **(F)** functional analysis of DR upregulated DEGs vs. DM by Metascape. **(G)** plasma levels of immunoglobulin subtypes were measured using a cytometric bead array assay. **(H)** heatmap of the selected diseases and functions by ingenuity pathway analysis across the DEGs of DM, DR, and DM–DR and Gene Expression Omnibus datasets of ocular tissues.

The infiltration and activation of immune cells, such as neutrophils, T cells, and B cells participate in the pathogenic process of DR (Pan et al., 2021). A total of 89 genes were found to be significantly upregulated and 128 genes were significantly downregulated in the DR group vs. the DM group whereas 44 upregulated genes and 153 downregulated genes in DM–DR vs. DM groups (Figure 5A, Supplementary Figure S5A,B). The majority of the significant DEGs were exclusively enriched in the comparison of DR vs. DM or that of DM–DR vs. DM and only 18 downregulated and 6 upregulated DEGs were shared among them (Figure 5B). The immune cell abundance identifier (ImmuCellAI) and Timer2 platforms were applied for the evaluation of the immune cell abundance from the RNA-seq data (Figure 5C). The percentages of B cells, CD4^+^ memory T cells, and T regulatory cells were higher in the DR group, whereas high abundances of NK cells, neutrophils, and M1 macrophages were observed in the DM–DR group. Besides cell abundance differences, T cell– and B cell–related functions were enhanced in patients with DR (DR in Figure 5D and DM–DR in Figure 5E) by GSEA analysis (Subramanian et al., 2005). GO and KEGG functional analyses were performed and, consistently, leukocyte activation and lymphocyte proliferation were upregulated in the DR group vs. the DM group (Figure 5F). The plasma immunoglobulin subtypes produced by B cells were measured, and the IgG1, IgG4, IgA, and IgD levels were increased in patients with DR especially DR patients (Figure 5G). Furthermore, the leukocyte proliferation and transmigration enriched in DR vs. DM comparison were also upregulated in the gene functions of ocular tissues with DR derived from GSE102485, GSE60436 (Ishikawa et al., 2015), and GSE94019 (Figure 5H). In accordance with the observed functional enrichment of significant DMGs in Figure 3, functions, such as cell adhesion, extracellular matrix structure, and neuronal system, were also enhanced in the upregulated DEGs of DR patients (Figure 5F). All these data suggested that pathogenic nonsynonymous gene mutations largely interfered with the immune cell abundance and functions to participate in the pathogenesis of DR.

### Genes and disease-function networks overlapped with ocular tissues of diabetic retinopathy

**FIGURE 6:**
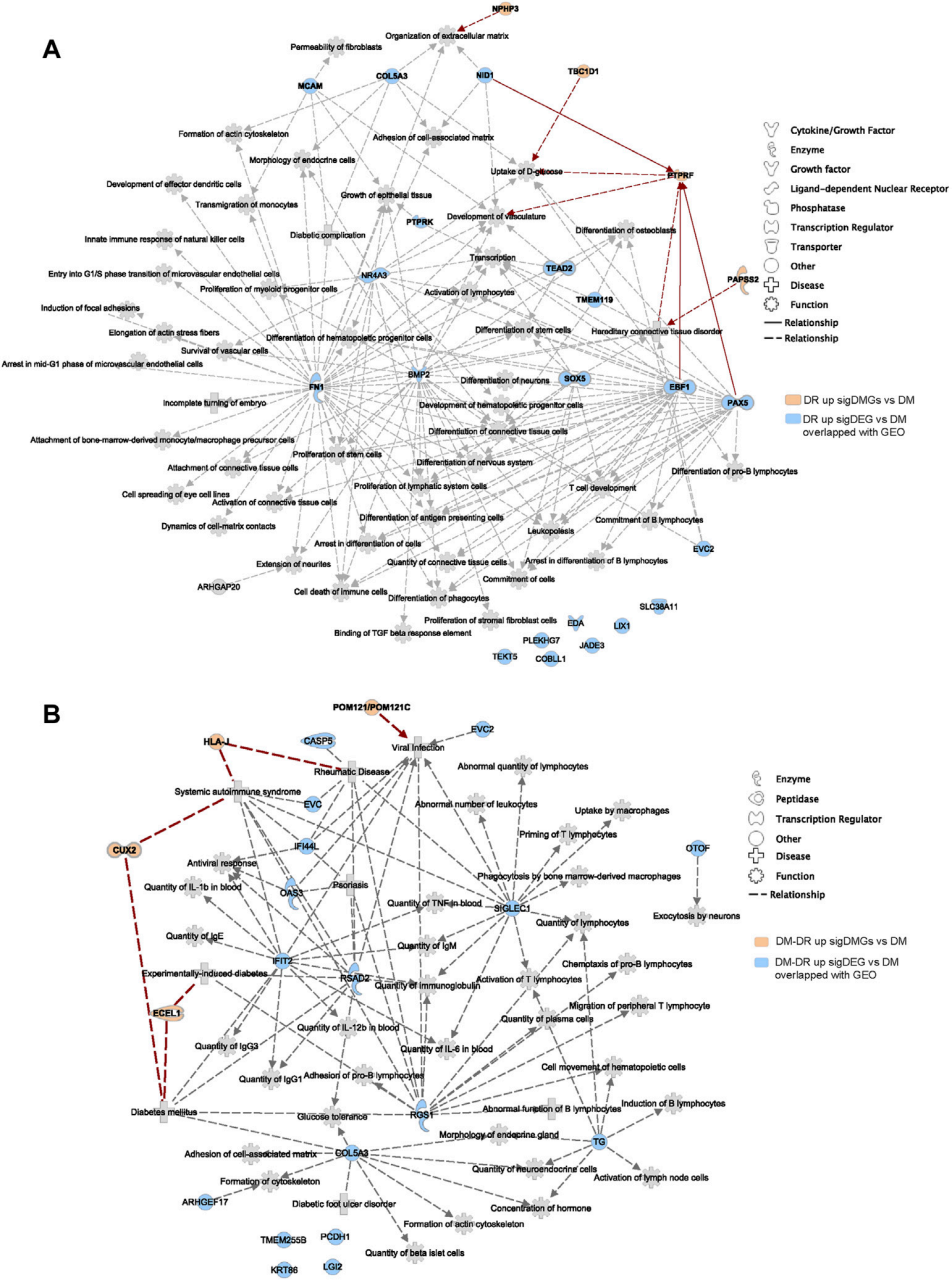
Disease and function network of upregulated differently expressed genes (DEGs) verified by Gene Expression Omnibus (GEO) data of patients with diabetic retinopathy (DR). **(A)** the disease and function analysis using ingenuity pathway analysis software of the overlapped upregulated DEGs of DR vs. non-DR (DM) and GEO datasets, including GSE102485, GSE94019, and GSE60436, and the direct and indirect interactions with the putatively susceptible differentially mutant genes (DMGs) of DR vs. DM. **(B)** the intersection analysis of the overlapped upregulated DEGs of DM–DR vs. DM with GEO datasets and the enriched DMGs of DM–DR vs. DM.

Immune cell infiltration and activation are tightly correlated with pathogenic changes in ocular tissues of patients with DR (Pan et al., 2021). As shown in Figure 5H, immune cell functional changes were verified by RNA-seq data of ocular tissues. We hypothesized that the upregulated DEGs of DR and DM–DR might be significantly increased in local eye tissues due to the immune cell infiltration and activation. A total of 21 genes were shared in the upregulated DEGs of DR vs. DM comparison and the upregulated DEGs derived from GSE102485, GSE60436, and GSE94019 datasets (Supplementary Figure S5). Disease and function network analysis connected these shared genes to immune cell proliferation, differentiation, and activation, as well as cytoskeleton changes, vascular development, glucose uptake, nervous system development, diabetic complication, and so on (Figure 6A). These genes, functions, and diseases were also directly or indirectly connected to the putatively susceptible DMGs of DR vs. DM (red, Figure 6A). Likewise, 17 upregulated DEGs of DM–DR vs. DM comparison overlapped with the upregulated DEGs of ocular tissues (Supplementary Figure S5C). The network of DMGs, DEGs, and their related functions and diseases was constructed using IPA, and diabetes mellitus, immune cell activation, immunoglobulin quantity, and other proinflammatory diseases and functions were enriched in the network (Figure 6B). In summary, a panel of the upregulated DEGs in DR or DM–DR groups were shared with ocular tissues, which participated in local immune cell function regulation and were tightly connected with certain putatively susceptible DMGs.

**FIGURE 7:**
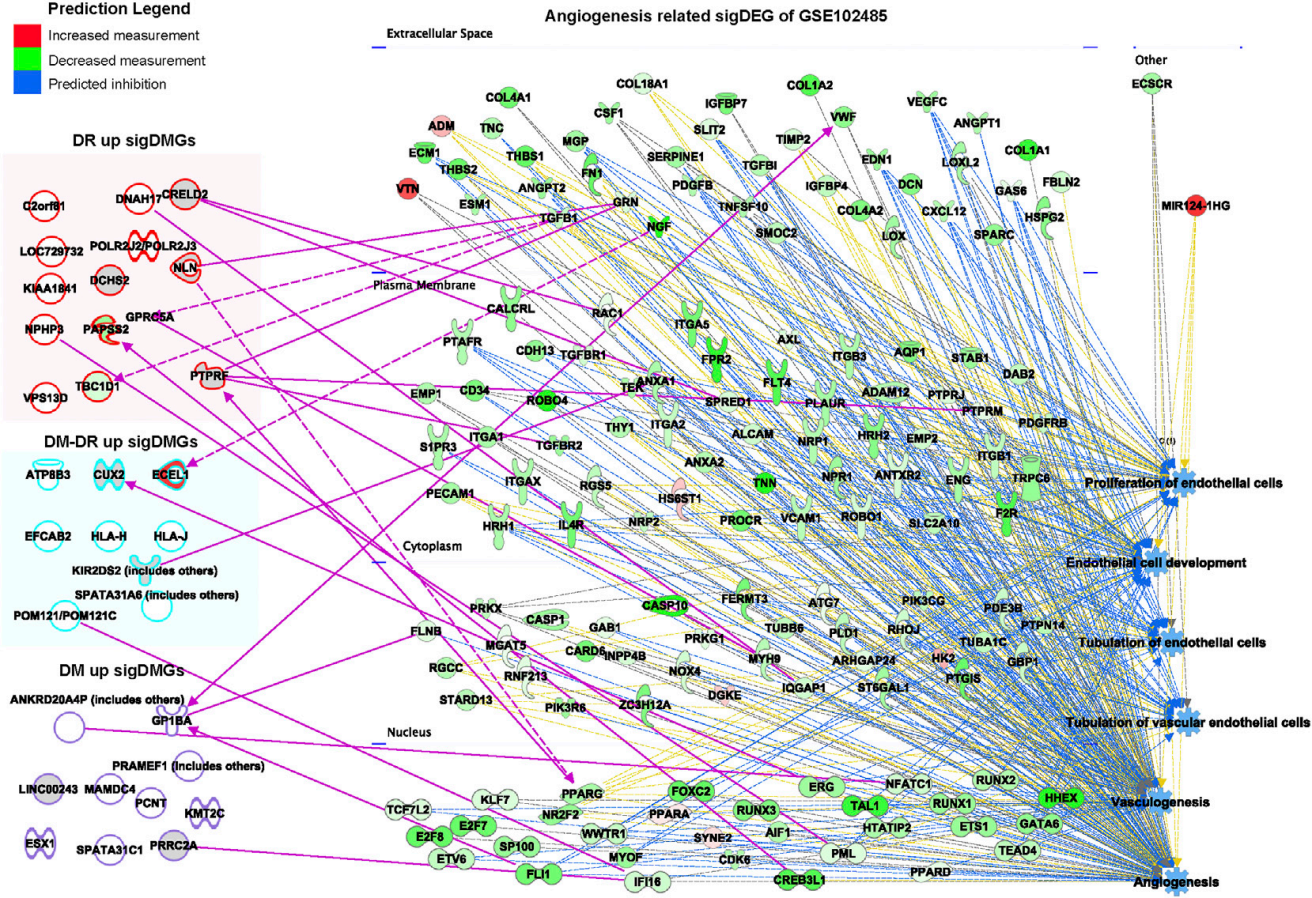
Network analysis of putatively susceptible or protective mutant genes with diabetic retinopathy–related upregulated genes enriched in the GSE102485 database. The summarized disease and function networks depict the interactions among the 54 significant differentially mutant genes with the angiogenesis-associated gene set from GSE102485. Green and red molecules indicate the upregulated and downregulated differently expressed genes of ocular tissues from patients with diabetic retinopathy vs. those without retinopathy, respectively. Figure legend displays gene and function symbol types and colors.

Pathogenic angiogenesis is the key feature for the occurrence of blinding diseases, especially DR. The ocular anti-VEGF treatment represents one of the most significant advancements in the treatment of DR (Cheung et al., 2014). The putatively susceptible and protective DMGs (Figure 2A) might also be involved in pathogenic angiogenesis. Function and disease analyses were performed for the significant DEGs of ocular tissues from DR patients vs. diabetic patients without retinopathy (GSE102485) using IPA software (Figure 7). The gene network associated with angiogenesis was identified and 12 putatively susceptible DMGs and three protective DMGs were predicted to be involved in the gene regulation of angiogenesis (Figure 7). These data suggested that the identified putatively pathogenic DMGs might also contribute to the pathogenic angiogenesis process of DR in addition to interfering with immune cell infiltration and functions.

## Discussion

DR is a leading cause of blindness in T2DM patients. Longer duration of disease, poor glycemic control, ineffective blood pressure control, and dyslipidemia are the well-recognized risk factors for DR. Accumulating evidence from recent studies, including twin studies, family studies, candidate gene studies, linkage studies, and small-scale GWAS studies, indicates that genetic variants may play an essential role in the pathogenesis of DR (Cho and Sobrin, 2014). In the present study, blood samples from three extreme phenotypes of T2DM patients were collected and processed for WES and RNA-seq analysis. In WES analysis, a total of 54 rare mutant genes were stringently filtered to represent the putative pathogenic or protective genes for DR occurrence, with various functions, including immune cell activation, glucose metabolism, renal abnormality, and nerve dysfunction. Through RNA-seq analysis, immune cell composition and activating status were connected to the DR pathology. Moreover, through integrative analysis with published transcriptomic data of ocular tissues of DR patients, a significant portion of DEGs and DMGs we identified in blood samples were able to map with local ocular tissue data, and functional analysis of these genes also suggested that both intraocular immune cell activation and angiogenesis were involved, indicating that immune function plays an important role in DR pathogenesis.

In previously reported studies (Cabrera et al., 2020), novel genetic variants *KLF17*, *ZNF395*, *CD33*, *PLEKHG5*, and *COL18A1* were identified in the “advanced” PDR cohort. These genes have been shown to be involved in the angiogenesis and inflammatory pathways, both implicated in DR progression. In our study, the identified putative pathogenic genes for DR patients were functionally enriched in ocular diseases and functions, activation of immune cells especially T cells, glucose metabolism, renal abnormality as well as nerve dysfunction. Besides, patients with early-onset retinopathy (DR) and late-onset retinopathy (DM–DR) carried different pathogenic mutations, pointing to the heterogeneity of DR pathogenesis. Among the 23 putatively pathogenic genes, approximately half of them were directly or indirectly connected to diabetic complications, especially DR. There was still a panel of mutant genes with unidentified connections with known diabetic terms. These data suggested that using the extreme phenotypic diabetic samples, novel genetic characteristics could be screened out to expand our understanding of DR pathogenesis. However, further studies need to be conducted to confirm the relationship of these newly identified mutant genes with DR *in vitro* and *in vivo*. According to our and others’ studies, the pathogenesis of DR might be simultaneously controlled or influenced by numerous gene mutations, which brings significant difficulties to select the optimal models to verify these findings. In fact, collecting more patient samples to verify and optimize the DMG panels might be an efficient approach. Post more strictly filtering in the large population studies, the number of valid DMGs could be decreased and theoretically, retinal organoids in corporation with a vascular network or retinal pigment epithelium as a-retina-on-a-chip format (Kruczek and Swaroop, 2020) might be used to verify the contribution of these mutations to DR to some extent. After key mutational events were verified, the remaining individual or limited gene mutations could be further investigated by diabetic mice or rat models (Olivares et al., 2017; Pitale and Gorbatyuk, 2022). For those DMGs linked to angiogenesis of DR, conventional 2D or advanced 3D coculture angiogenesis models could also be applied to analyze the relationship of these gene mutations with angiogenesis of DR (Citi et al., 2020; Eyre et al., 2020).

Besides the mutations in the protein-coding region, several mutations within noncoding regions of the genome, such as intron polymorphism of *NVL*, *eNOS*, and *ACE* (Bhatwadekar et al., 2021), were previously reported to be associated with DR pathogenesis. Risch and Merikangas proposed that genetic variants located in intronic or intergenic regions, which were associated with DR, mostly appeared to play important functional roles in regulating gene expression (Skol et al., 2020). More genomic variants in noncoding regions related to DR pathogenesis might be identified using whole-genome sequencing instead of WES, even though some noncoding genomic mutations could also be detected in WES (Naruto et al., 2015).

Angiogenesis is the physiologic condition characterized by the growth of new blood vessels originating from preexisting ones. New vessel formation in patients with DR causes vision loss and is the main target of treatment for this condition (Yang et al., 2020). Numerous angiogenic factors have been implicated in the pathogenesis of DR. Considering the key role of angiogenesis in DR development and progression, we analyzed the significant DEGs of ocular tissues of DR patients vs. diabetic patients without retinopathy and identified the gene network associated with angiogenesis. Twelve putatively susceptible DMGs and three protective DMGs were found to be correlated with the DR-related angiogenesis gene network. These data suggested that the DMGs we identified using blood samples (mainly for germline mutations) could at least partially predict the mutation-mediated functional dysregulation within eye tissues. Even though the exact roles of those DMGs involved in angiogenesis or DR mechanism need to be determined in further detailed experiments, blood sample-based exome/RNA-seq analysis was valuable for clinical DR prediction due to the easier accessibility of blood samples. In addition to their connection with the angiogenesis of DR, those DMGs we identified were also partially verified by the genomic variants derived from the patient samples collected in the United States (GSE94019). Therefore, we could speculate that those putatively susceptible and protective mutant genes identified in the Han population could possibly be extended to the pathogenic prediction of other ethnic groups. However, further studies with DR samples from different ethnic groups are needed to verify this hypothesis.

Limitations exist in our research due to the relatively small sample size. Larger scale studies are needed to verify these 54 gene mutations susceptible to DR, including DM, DR, and DM–DR subgroups. As observed by the clinicians, there is a population of well-controlled diabetics who nonetheless will develop persistent severe retinopathy and diabetic macular edema. It is worth finding out whether pathogenic mutational events and/or high glucose levels are the dominant causes of DR occurrence. Future studies should be performed to compare the putative mutations between severe DR and DM patients in the context of well-controlled glycemic levels, by which the DR pathogenic factors independent of glycemia could be identified. At least, our preliminary study shed a light on the potential usage of germline mutations to warn T2DM individuals with a high risk of DR to take regular ophthalmological examinations.

## Data Availability

The datasets presented in this study can be found in online repositories. The name of the repository and accession number can be found below: GSA for Human (National Genomics Data Center); HRA002375.

## References

[B1] Alghadyan A. A. (2011). Diabetic retinopathy - an update. Saudi J. Ophthalmol. 25 (2), 99–111. 10.1016/j.sjopt.2011.01.009 23960911 PMC3729572

[B2] American Diabetes Association (2020). 2. Classification and diagnosis of diabetes: Standards of medical care in diabetes-2020. Diabetes Care 43, S14–s31. 10.2337/dc20-S002 31862745

[B3] Anders S. Pyl P. T. Huber W. (2015). HTSeq--a Python framework to work with high-throughput sequencing data. Bioinformatics 31 (2), 166–169. 10.1093/bioinformatics/btu638 25260700 PMC4287950

[B4] Arar N. H. Freedman B. I. Adler S. G. Iyengar S. K. Chew E. Y. Davis M. D. (2008). Heritability of the severity of diabetic retinopathy: The FIND-eye study. Invest. Ophthalmol. Vis. Sci. 49 (9), 3839–3845. 10.1167/iovs.07-1633 18765632 PMC2583147

[B5] Bhatwadekar A. D. Shughoury A. Belamkar A. Ciulla T. A. (2021). Genetics of diabetic retinopathy, a leading cause of irreversible blindness in the industrialized world. Genes. (Basel) 12 (8), 1200. 10.3390/genes12081200 34440374 PMC8394456

[B6] Cabrera A. P. Mankad R. N. Marek L. Das R. Rangasamy S. Monickaraj F. (2020). Genotypes and phenotypes: A search for influential genes in diabetic retinopathy. Int. J. Mol. Sci. 21 (8), E2712. 10.3390/ijms21082712 32295293 PMC7215289

[B7] Chaudhury A. Duvoor C. Reddy Dendi V. S. Kraleti S. Chada A. Ravilla R. (2017). Clinical review of antidiabetic drugs: Implications for type 2 diabetes mellitus management. Front. Endocrinol. 8, 6. 10.3389/fendo.2017.00006 PMC5256065 28167928

[B8] Cheung N. Mitchell P. Wong T. Y. (2010). Diabetic retinopathy. Lancet 376 (9735), 124–136. 10.1016/S0140-6736(09)62124-3 20580421

[B9] Cheung N. Wong I. Y. Wong T. Y. (2014). Ocular anti-VEGF therapy for diabetic retinopathy: Overview of clinical efficacy and evolving applications. Diabetes Care 37 (4), 900–905. 10.2337/dc13-1990 24652721

[B10] Cho H. Sobrin L. (2014). Genetics of diabetic retinopathy. Curr. Diab. Rep. 14 (8), 515. 10.1007/s11892-014-0515-z 24952107 PMC4125976

[B11] Citi V. Piragine E. Brogi S. Ottino S. Calderone V. (2020). Development of *in vitro* corneal models: Opportunity for pharmacological testing. Methods Protoc. 3 (4), E74. 10.3390/mps3040074 33147693 PMC7711486

[B12] Cole J. B. Florez J. C. (2020). Genetics of diabetes mellitus and diabetes complications. Nat. Rev. Nephrol. 16 (7), 377–390. 10.1038/s41581-020-0278-5 32398868 PMC9639302

[B13] Ebneter A. Zinkernagel M. S. (2016). Novelties in diabetic retinopathy. Endocr. Dev. 31, 84–96. 10.1159/000439391 26824524

[B14] Eyre J. J. Williams R. L. Levis H. J. (2020). A human retinal microvascular endothelial-pericyte co-culture model to study diabetic retinopathy *in vitro* . Exp. Eye Res. 201, 108293. 10.1016/j.exer.2020.108293 33039459

[B15] Fong D. S. Aiello L. P. Ferris F. L. Klein R. (2004). Diabetic retinopathy. Diabetes Care 27 (10), 2540–2553. 10.2337/diacare.27.10.2540 15451934

[B16] Gella L. Raman R. Kulothungan V. Pal S. S. Ganesan S. Srinivasan S. (2017). Color vision abnormalities in type II diabetes: Sankara nethralaya diabetic retinopathy epidemiology and molecular genetics study II report no 2. Indian J. Ophthalmol. 65 (10), 989–994. 10.4103/ijo.IJO_601_16 29044066 PMC5678337

[B17] Hietala K. Forsblom C. Summanen P. Groop P. H. (2008). Heritability of proliferative diabetic retinopathy. Diabetes 57 (8), 2176–2180. 10.2337/db07-1495 18443200 PMC2494680

[B18] Hirsch I. B. Brownlee M. (2010). Beyond hemoglobin A1c--need for additional markers of risk for diabetic microvascular complications. Jama 303 (22), 2291–2292. 10.1001/jama.2010.785 20530784

[B19] Ishikawa K. Yoshida S. Kobayashi Y. Zhou Y. Nakama T. Nakao S. (2015). Microarray analysis of gene expression in fibrovascular membranes excised from patients with proliferative diabetic retinopathy. Invest. Ophthalmol. Vis. Sci. 56 (2), 932–946. 10.1167/iovs.14-15589 25604687

[B20] Jenkins A. J. Joglekar M. V. Hardikar A. A. Keech A. C. O'Neal D. N. Januszewski A. S. (2015). Biomarkers in diabetic retinopathy. Rev. Diabet. Stud. 12 (1-2), 159–195. 10.1900/RDS.2015.12.159 26676667 PMC5397989

[B21] Kaur N. Vanita V. (2016). Association of aldose reductase gene (AKR1B1) polymorphism with diabetic retinopathy. Diabetes Res. Clin. Pract. 121, 41–48. 10.1016/j.diabres.2016.08.019 27640118

[B22] Kim D. Langmead B. Salzberg S. L. (2015). HISAT: A fast spliced aligner with low memory requirements. Nat. Methods 12 (4), 357–360. 10.1038/nmeth.3317 25751142 PMC4655817

[B23] Krämer A. Green J. Pollard J. Jr. Tugendreich S. (2014). Causal analysis approaches in ingenuity pathway analysis. Bioinformatics 30 (4), 523–530. 10.1093/bioinformatics/btt703 24336805 PMC3928520

[B24] Kruczek K. Swaroop A. (2020). Pluripotent stem cell-derived retinal organoids for disease modeling and development of therapies. Stem Cells 38 (10), 1206–1215. 10.1002/stem.3239 32506758 PMC7586922

[B25] Lam J. D. Oh D. J. Wong L. L. Amarnani D. Park-Windhol C. Sanchez A. V. (2017). Identification of RUNX1 as a mediator of aberrant retinal angiogenesis. Diabetes 66 (7), 1950–1956. 10.2337/db16-1035 28400392 PMC5482092

[B26] Li Y. Chen D. Sun L. Wu Y. Zou Y. Liang C. (2019). Induced expression of VEGFC, ANGPT, and EFNB2 and their receptors characterizes neovascularization in proliferative diabetic retinopathy. Invest. Ophthalmol. Vis. Sci. 60 (13), 4084–4096. 10.1167/iovs.19-26767 31574534

[B27] Lin Y. Xiao Y. C. Zhu H. Xu Q. Y. Qi L. Wang Y. B. (2014). Serum fibroblast growth factor 21 levels are correlated with the severity of diabetic retinopathy. J. Diabetes Res. 2014, 929756. 10.1155/2014/929756 24895642 PMC4009259

[B28] Mertens F. Johansson B. Fioretos T. Mitelman F. (2015). The emerging complexity of gene fusions in cancer. Nat. Rev. Cancer 15 (6), 371–381. 10.1038/nrc3947 25998716

[B29] Naruto T. Okamoto N. Masuda K. Endo T. Hatsukawa Y. Kohmoto T. (2015). Deep intronic GPR143 mutation in a Japanese family with ocular albinism. Sci. Rep. 5, 11334. 10.1038/srep11334 26061757 PMC4650666

[B30] Olivares A. M. Althoff K. Chen G. F. Wu S. Morrisson M. A. DeAngelis M. M. (2017). Animal models of diabetic retinopathy. Curr. Diab. Rep. 17 (10), 93. 10.1007/s11892-017-0913-0 28836097 PMC5569142

[B31] Pan W. W. Lin F. Fort P. E. (2021). The innate immune system in diabetic retinopathy. Prog. Retin. Eye Res. 84, 100940. 10.1016/j.preteyeres.2021.100940 33429059 PMC8263813

[B32] Piskol R. Ramaswami G. Li J. B. (2013). Reliable identification of genomic variants from RNA-seq data. Am. J. Hum. Genet. 93 (4), 641–651. 10.1016/j.ajhg.2013.08.008 24075185 PMC3791257

[B33] Pitale P. M. Gorbatyuk M. S. (2022). Diabetic retinopathy: From animal models to cellular signaling. Int. J. Mol. Sci. 23 (3), 1487. 10.3390/ijms23031487 35163410 PMC8835767

[B34] Roberts A. Pimentel H. Trapnell C. Pachter L. (2011). Identification of novel transcripts in annotated genomes using RNA-Seq. Bioinformatics 27 (17), 2325–2329. 10.1093/bioinformatics/btr355 21697122

[B35] Shtir C. Aldahmesh M. A. Al-Dahmash S. Abboud E. Alkuraya H. Abouammoh M. A. (2016). Exome-based case-control association study using extreme phenotype design reveals novel candidates with protective effect in diabetic retinopathy. Hum. Genet. 135 (2), 193–200. 10.1007/s00439-015-1624-8 26693933

[B36] Skol A. D. Jung S. C. Sokovic A. M. Chen S. Fazal S. Sosina O. (2020). Integration of genomics and transcriptomics predicts diabetic retinopathy susceptibility genes. Elife 9, e59980. 10.7554/eLife.59980 33164750 PMC7728435

[B37] Stratton I. M. Kohner E. M. Aldington S. J. Turner R. C. Holman R. R. Manley S. E. (2001). Ukpds 50: Risk factors for incidence and progression of retinopathy in type II diabetes over 6 years from diagnosis. Diabetologia 44 (2), 156–163. 10.1007/s001250051594 11270671

[B38] Subramanian A. Tamayo P. Mootha V. K. Mukherjee S. Ebert B. L. Gillette M. A. (2005). Gene set enrichment analysis: A knowledge-based approach for interpreting genome-wide expression profiles. Proc. Natl. Acad. Sci. U. S. A. 102 (43), 15545–15550. 10.1073/pnas.0506580102 16199517 PMC1239896

[B39] Sun Z. Bhagwate A. Prodduturi N. Yang P. Kocher J. A. (2017). Indel detection from RNA-seq data: Tool evaluation and strategies for accurate detection of actionable mutations. Brief. Bioinform. 18 (6), 973–983. 10.1093/bib/bbw069 27473065 PMC5862335

[B40] ValdezGuerrero A. S. Quintana-Pérez J. C. Arellano-Mendoza M. G. Castañeda-Ibarra F. J. Tamay-Cach F. Alemán-González-Duhart D. (2021). Diabetic retinopathy: Important biochemical alterations and the main treatment strategies. Can. J. Diabetes 45 (6), 504–511. 10.1016/j.jcjd.2020.10.009 33341391

[B41] Wang K. Li M. Hakonarson H. (2010). Annovar: Functional annotation of genetic variants from high-throughput sequencing data. Nucleic Acids Res. 38 (16), e164. 10.1093/nar/gkq603 20601685 PMC2938201

[B42] Whitehead M. Wickremasinghe S. Osborne A. Van Wijngaarden P. Martin K. R. (2018). Diabetic retinopathy: A complex pathophysiology requiring novel therapeutic strategies. Expert Opin. Biol. Ther. 18 (12), 1257–1270. 10.1080/14712598.2018.1545836 30408422 PMC6299358

[B43] Wilkinson C. P. Ferris F. L. 3rd Klein R. E. Lee P. P. Agardh C. D. Davis M. (2003). Proposed international clinical diabetic retinopathy and diabetic macular edema disease severity scales. Ophthalmology 110 (9), 1677–1682. 10.1016/S0161-6420(03)00475-5 13129861

[B44] Wong T. Y. Cheung C. M. G. Larsen M. Sharma S. Simo R. (2016). Diabetic retinopathy. Nat. Rev. Dis. Prim. 2, 16012. 10.1038/nrdp.2016.12 27159554

[B45] Xu H. Chen M. (2017). Diabetic retinopathy and dysregulated innate immunity. Vis. Res. 139, 39–46. 10.1016/j.visres.2017.04.013 28571700

[B46] Yang Y. Liu Y. Li Y. Chen Z. Xiong Y. Zhou T. (2020). MicroRNA-15b targets VEGF and inhibits angiogenesis in proliferative diabetic retinopathy. J. Clin. Endocrinol. Metab. 105 (11), dgaa538. 10.1210/clinem/dgaa538 32797181 PMC7947967

[B47] Zheng Y. He M. Congdon N. (2012). The worldwide epidemic of diabetic retinopathy. Indian J. Ophthalmol. 60 (5), 428–431. 10.4103/0301-4738.100542 22944754 PMC3491270

[B48] Zhou Y. Zhou B. Pache L. Chang M. Khodabakhshi A. H. Tanaseichuk O. (2019). Metascape provides a biologist-oriented resource for the analysis of systems-level datasets. Nat. Commun. 10 (1), 1523. 10.1038/s41467-019-09234-6 30944313 PMC6447622

