# Supplementary material for: Identifying gene variants underlying the pathogenesis of diabetic retinopathy based on integrated genomic and transcriptomic analysis of clinical extreme phenotypes
Source: Front Genet. 2022 Aug 10;13:929049. doi: 10.3389/fgene.2022.929049 (PMC9399422; doi:10.3389/fgene.2022.929049)
Supplement: Supplementary file 1 [file DataSheet1.DOCX]

Supplementary Material


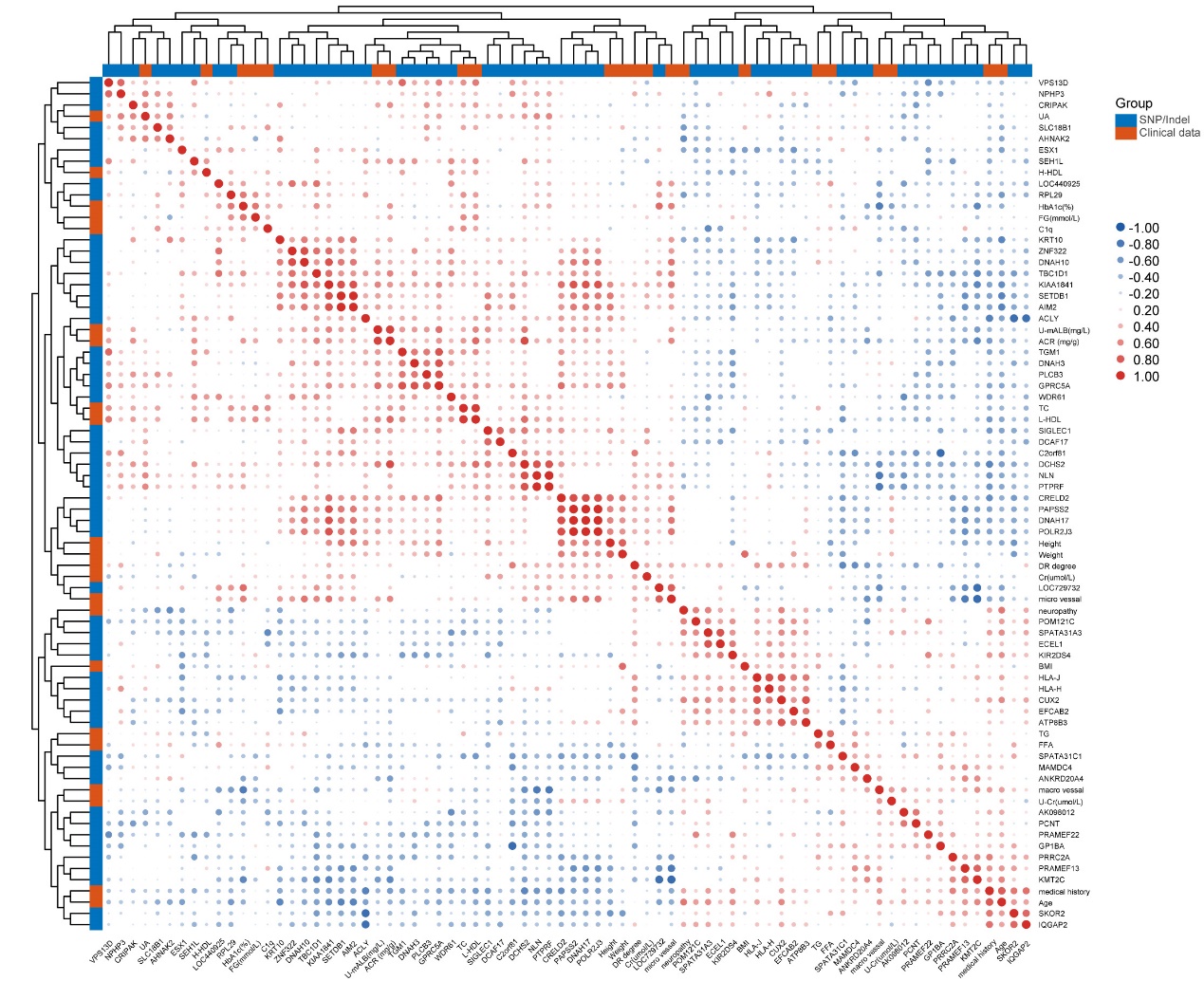


**Supplementary Figure 1.** Pearson correlation between putatively damaging or protecting mutant genes and clinical profile data.


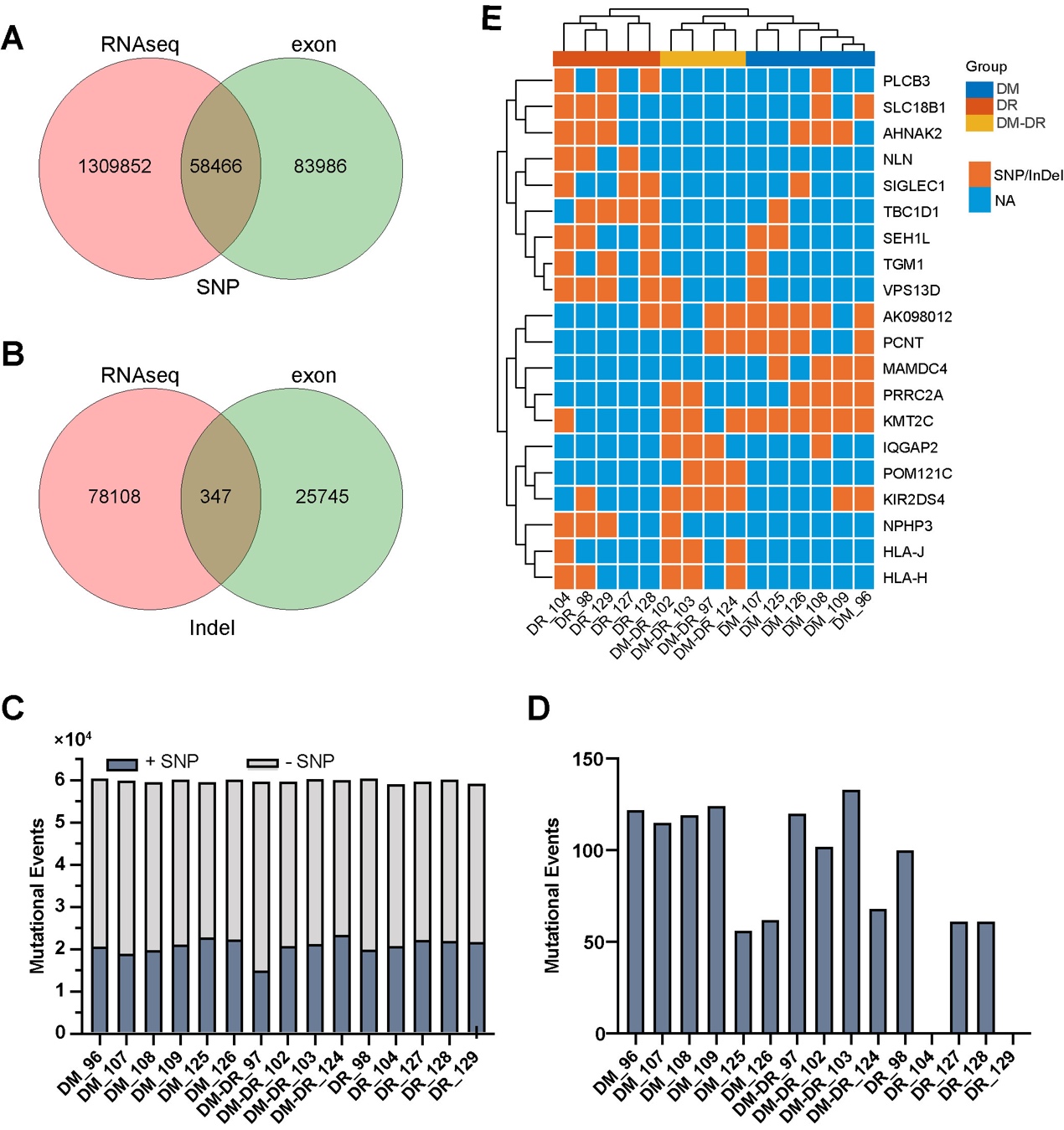


**Supplementary Figure 2.** Putatively damaging or protecting mutant genes were verified by RNA-seq mutational data. **(A-B)** SNP (A) and Indel (B) variants of exome sequencing were validated by RNA-seq data. **(C-D)** The numbers of mutational events of exome sequencing were verified by RNA-seq data within each sample. **(E)** Heat map-based hierarchical clustering of the 20 out of 54 putatively pathogenic DMGs identified by WES were verified by RNA-seq. They were separated into these 3 groups. Under each cluster, the samples were sub-grouped by their patients of origin.


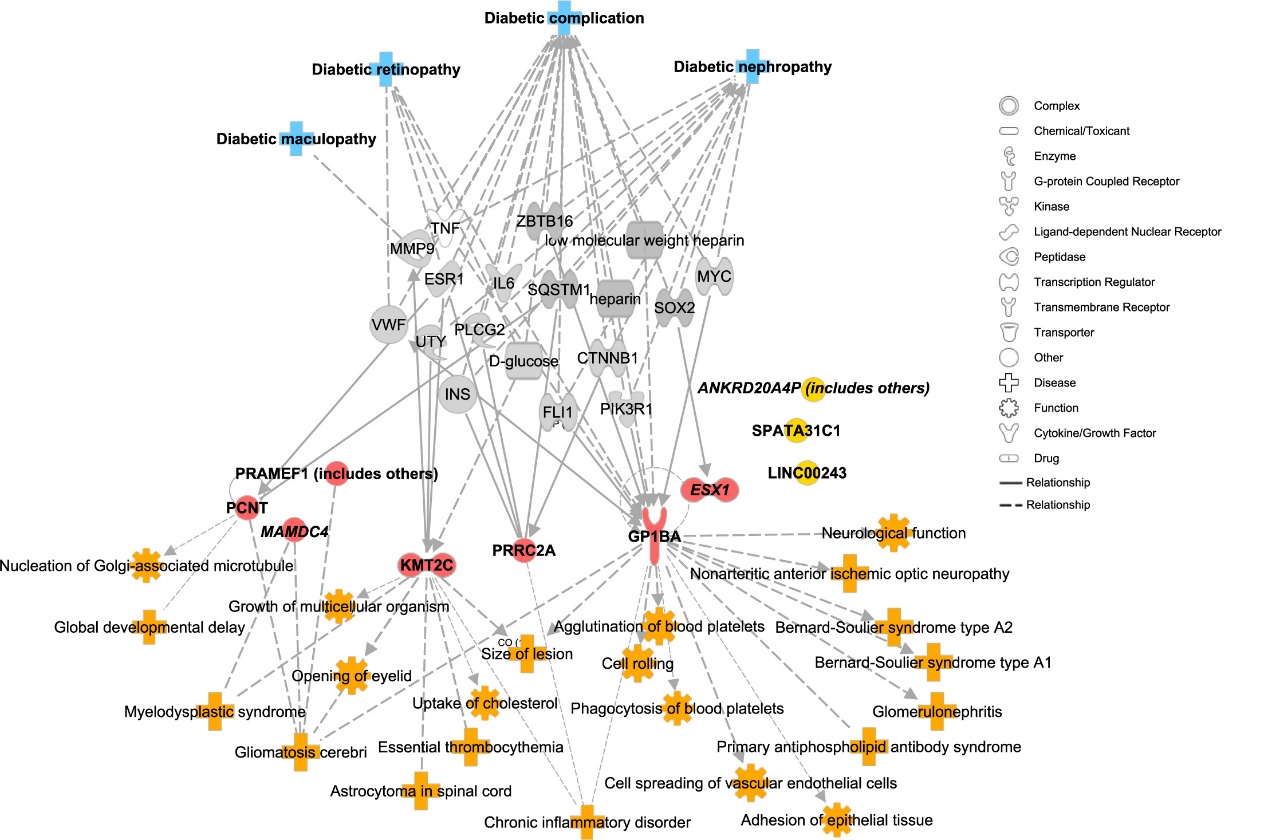


**Supplementary Figure 3.** Network analysis of putatively pathogenic mutant genes enriched in patients without oculopathy for at least 10 years.


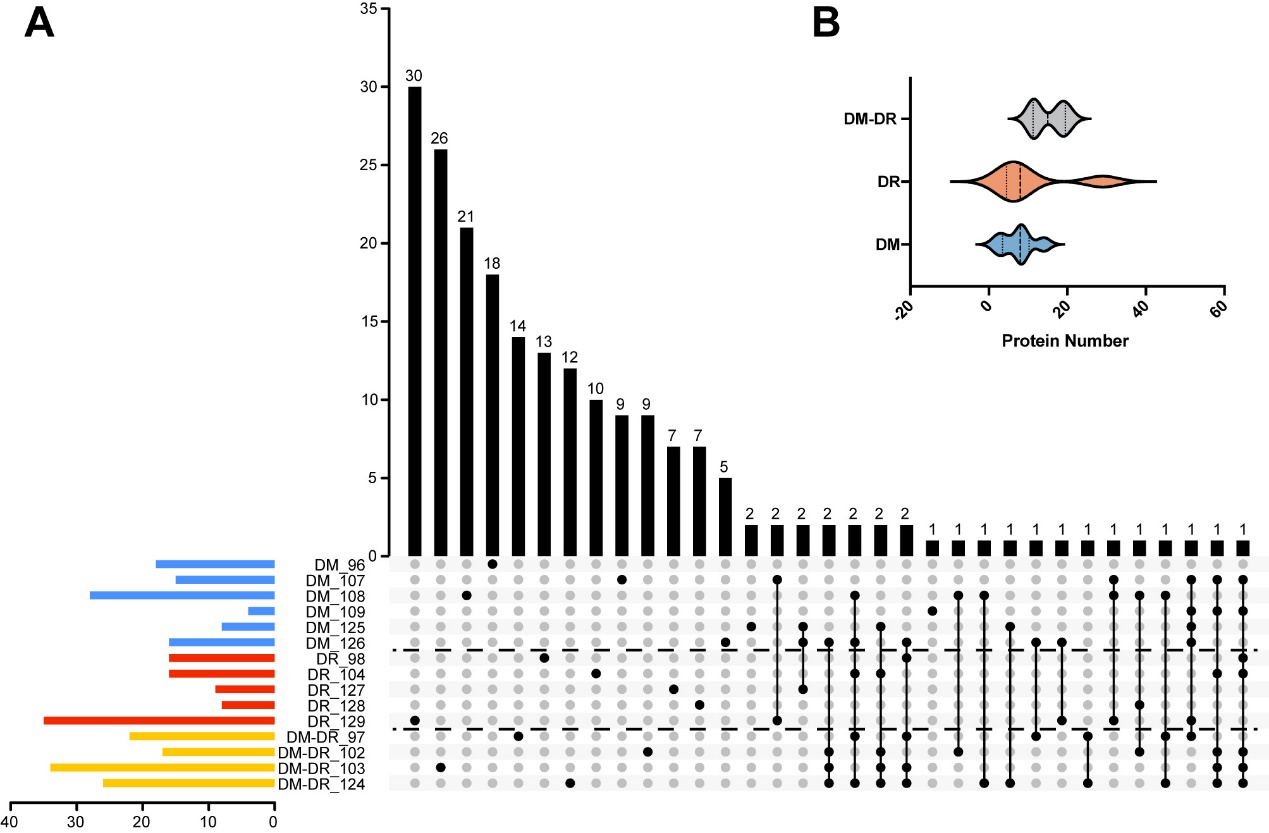


**Supplementary Figure 4.** Fusion gene analysis across groups. **(A)** The genes in fusion genes were compared across all the sample from 3 groups. **(B)** The fusion gene number in each group.


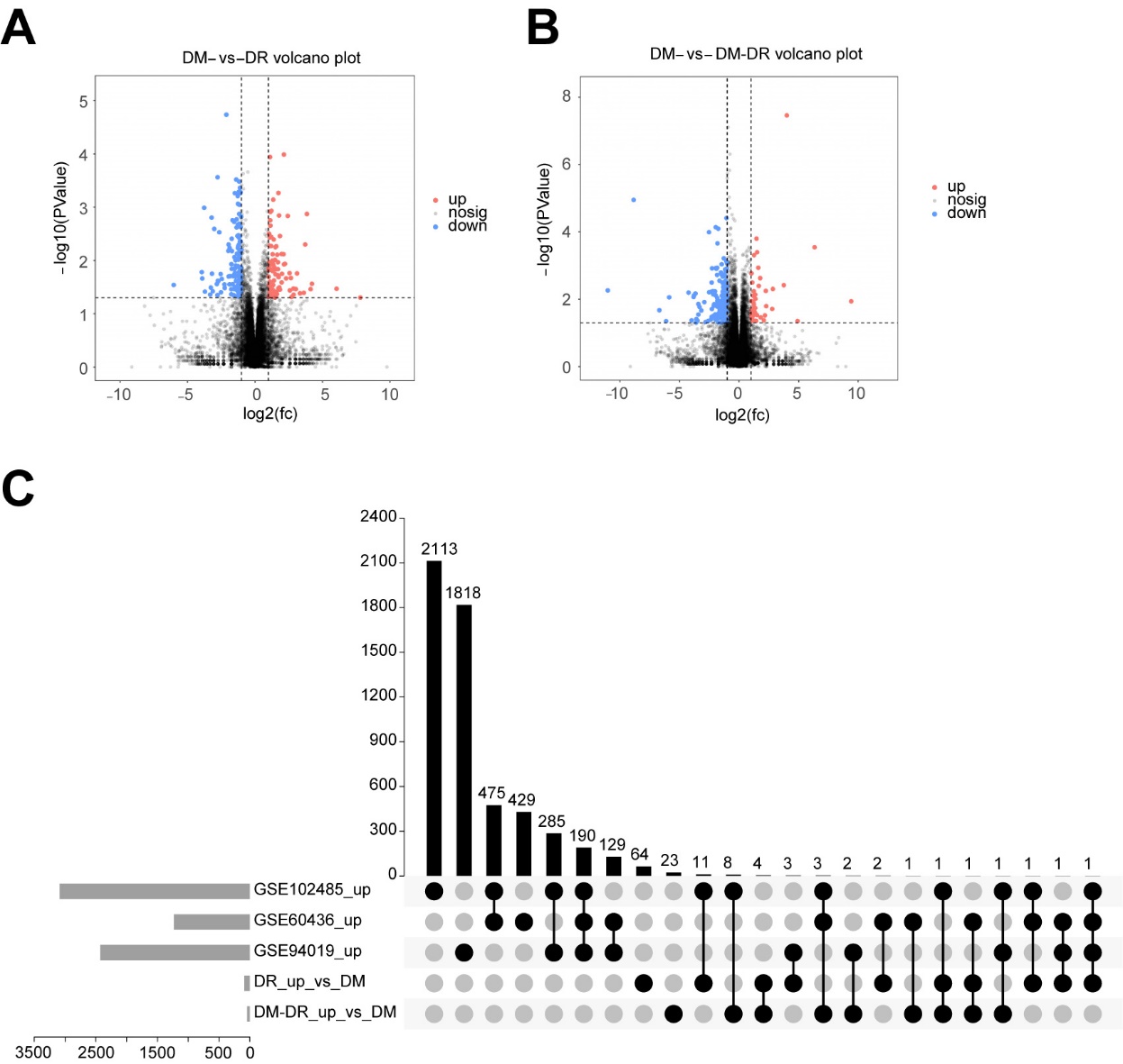


**Supplementary Figure 5.** Transcriptome profiles across three groups. **(A)** The volcano plot of DEGs between DM-vs-DR groups. The grey dots represented genes without significant difference between two groups, the red dots showed those genes’ expression level was significantly up-regulated and the blue dots mean significantly down-regulated in DR groups, compared with DM group. **(B)** The volcano plot of DEGs between DM-vs-DM-DR groups. The meaning of plots was same as (A). **(C)** UpSet plot showing the intersection of the upregulated DEGs of DR vs DM and DM-DR vs DM overlapped with upregulated DEGs of ocular tissues in GSE102485, GSE60436 and GSE94019.

**Supplementary Table1. Detailed gene mutation (SNP indel list of 54 sigDMGs).**

| Chr | Start | End | Ref | Alt | variation | database | Symbol | Function |
| --- | --- | --- | --- | --- | --- | --- | --- | --- |
| chr1 | 12320753 | 12320753 | G | A | SNP | rs76782506 | VPS13D | exonic nonsynonymous SNV |
| chr1 | 12337820 | 12337820 | C | T | SNP | rs140179355 | VPS13D | exonic nonsynonymous SNV |
| chr1 | 12337888 | 12337888 | G | A | SNP | rs79282181 | VPS13D | exonic nonsynonymous SNV |
| chr1 | 12371668 | 12371668 | G | C | SNP | . | VPS13D | exonic nonsynonymous SNV |
| chr1 | 12401932 | 12401932 | A | T | SNP | rs184253678 | VPS13D | exonic nonsynonymous SNV |
| chr1 | 13036247 | 13036247 | T | A | SNP | rs71279237 | PRAMEF22 | exonic nonsynonymous SNV |
| chr1 | 13038119 | 13038119 | G | C | SNP | . | PRAMEF22 | exonic nonsynonymous SNV |
| chr1 | 13448180 | 13448180 | A | G | SNP | . | PRAMEF13 | exonic nonsynonymous SNV |
| chr1 | 13448184 | 13448184 | C | T | SNP | . | PRAMEF13 | exonic nonsynonymous SNV |
| chr1 | 44044864 | 44044864 | C | T | SNP | rs142335790 | PTPRF | exonic nonsynonymous SNV |
| chr1 | 44063508 | 44063508 | C | T | SNP | rs17849101 | PTPRF | exonic nonsynonymous SNV |
| chr1 | 44071946 | 44071948 | GCG | - | Deletion | rs777273194 | PTPRF | exonic nonframeshift deletion |
| chr1 | 150933562 | 150933562 | A | T | SNP | . | SETDB1 | exonic nonsynonymous SNV |
| chr1 | 150933570 | 150933570 | A | T | SNP | . | SETDB1 | exonic nonsynonymous SNV |
| chr1 | 150933575 | 150933575 | C | G | SNP | . | SETDB1 | exonic nonsynonymous SNV |
| chr1 | 159032486 | 159032486 | - | T | Insertion | rs554845943 | AIM2 | exonic frameshift insertion |
| chr1 | 245133569 | 245133569 | - | ACCCCCCCCCCCCC | Insertion | . | EFCAB2 | exonic frameshift insertion |
| chr1 | 245133569 | 245133569 | - | C | Insertion | . | EFCAB2 | exonic frameshift insertion |
| chr1 | 245133584 | 245133584 | G | C | SNP | . | EFCAB2 | exonic nonsynonymous SNV |
| chr1 | 245133588 | 245133588 | A | C | SNP | . | EFCAB2 | exonic nonsynonymous SNV |
| chr10 | 89473069 | 89473069 | - | AAAAAAAAAAAAAAAA | Insertion | . | PAPSS2 | splicing |
| chr10 | 89473069 | 89473069 | - | AAAAAA | Insertion | . | PAPSS2 | splicing |
| chr10 | 89473069 | 89473069 | - | AAAAA | Insertion | rs367885911 | PAPSS2 | splicing |
| chr10 | 89473069 | 89473069 | - | AAAA | Insertion | rs367885911 | PAPSS2 | splicing |
| chr10 | 89473069 | 89473069 | - | A | Insertion | rs367885911 | PAPSS2 | splicing |
| chr11 | 64032495 | 64032495 | A | - | Deletion | . | PLCB3 | exonic frameshift deletion |
| chr11 | 64032501 | 64032502 | AG | - | Deletion | . | PLCB3 | exonic frameshift deletion |
| chr11 | 64032505 | 64032516 | CACTGGATGCCT | - | Deletion | . | PLCB3 | exonic nonframeshift deletion |
| chr11 | 64032506 | 64032523 | ACTGGATGCCTCCCCCCG | - | Deletion | . | PLCB3 | exonic nonframeshift deletion |
| chr11 | 64032506 | 64032516 | ACTGGATGCCT | - | Deletion | . | PLCB3 | exonic frameshift deletion |
| chr11 | 64032522 | 64032522 | - | AG | Insertion | . | PLCB3 | exonic frameshift insertion |
| chr11 | 64032525 | 64032525 | - | A | Insertion | . | PLCB3 | exonic frameshift insertion |
| chr11 | 64032784 | 64032784 | G | A | SNP | rs79573066 | PLCB3 | exonic nonsynonymous SNV |
| chr12 | 13061553 | 13061553 | C | T | SNP | rs76634522 | GPRC5A | exonic nonsynonymous SNV |
| chr12 | 111785422 | 111785422 | - | TC | Insertion | . | CUX2 | exonic frameshift insertion |
| chr12 | 111785423 | 111785425 | CCC | - | Deletion | . | CUX2 | exonic nonframeshift deletion |
| chr12 | 111785430 | 111785431 | AG | - | Deletion | . | CUX2 | exonic frameshift deletion |
| chr12 | 124247077 | 124247077 | A | C | SNP | rs763635181 | DNAH10 | exonic nonsynonymous SNV |
| chr12 | 124325949 | 124325951 | AGG | - | Deletion | . | DNAH10 | exonic nonframeshift deletion |
| chr12 | 124333344 | 124333344 | G | A | SNP | rs369904884 | DNAH10 | exonic nonsynonymous SNV |
| chr12 | 124399076 | 124399076 | C | T | SNP | rs182386169 | DNAH10 | exonic nonsynonymous SNV |
| chr14 | 24731026 | 24731026 | T | C | SNP | rs138592626 | TGM1 | exonic nonsynonymous SNV |
| chr14 | 105407525 | 105407525 | G | A | SNP | rs76611205 | AHNAK2 | exonic nonsynonymous SNV |
| chr14 | 105411346 | 105411346 | G | A | SNP | rs752292717 | AHNAK2 | exonic nonsynonymous SNV |
| chr14 | 105412805 | 105412805 | T | C | SNP | rs753472154 | AHNAK2 | exonic nonsynonymous SNV |
| chr14 | 105413791 | 105413791 | G | A | SNP | rs57308399 | AHNAK2 | exonic nonsynonymous SNV |
| chr14 | 105414676 | 105414676 | G | A | SNP | rs528075601 | AHNAK2 | exonic nonsynonymous SNV |
| chr14 | 105415933 | 105415933 | G | A | SNP | rs79599750 | AHNAK2 | exonic nonsynonymous SNV |
| chr14 | 105419610 | 105419610 | C | G | SNP | rs117226478 | AHNAK2 | exonic nonsynonymous SNV |
| chr15 | 78587743 | 78587743 | - | TT | Insertion | rs34232388 | WDR61 | exonic frameshift insertion |
| chr15 | 78587744 | 78587747 | TTTT | - | Deletion | . | WDR61 | exonic frameshift deletion |
| chr15 | 78587744 | 78587746 | TTT | - | Deletion | rs756697782 | WDR61 | exonic nonframeshift deletion |
| chr15 | 78587744 | 78587744 | - | T | Insertion | . | WDR61 | exonic frameshift insertion |
| chr15 | 78587745 | 78587745 | - | T | Insertion | . | WDR61 | exonic frameshift insertion |
| chr15 | 78587746 | 78587746 | - | T | Insertion | . | WDR61 | exonic frameshift insertion |
| chr16 | 20966291 | 20966291 | G | T | SNP | rs34771199 | DNAH3 | exonic nonsynonymous SNV |
| chr16 | 20975540 | 20975540 | G | C | SNP | rs770265073 | DNAH3 | exonic nonsynonymous SNV |
| chr16 | 20975817 | 20975817 | A | G | SNP | rs146749118 | DNAH3 | exonic nonsynonymous SNV |
| chr16 | 21014485 | 21014485 | - | ATG | Insertion | rs755029918 | DNAH3 | exonic nonframeshift insertion |
| chr17 | 4837118 | 4837195 | AGCCCGACCACCCCAGAGCCCACCTCAGAGCCCGCCCCCAGCCCGACCACCCCGGAGCCCACCTCAGAGCCCGCCCCC | - | Deletion | . | GP1BA | exonic nonframeshift deletion |
| chr17 | 38975103 | 38975103 | - | GCTGCCGCCGCCGTATCCGCCGCCGGAGCTGCTGCCGCCGCCGTATCCGCCGCCGGAGCT | Insertion | . | KRT10 | exonic nonframeshift insertion |
| chr17 | 38975103 | 38975103 | - | GCTGCCGCCGCCGTATCCGCCGCCGGAGCT | Insertion | rs776920005 | KRT10 | exonic nonframeshift insertion |
| chr17 | 38975327 | 38975327 | T | - | Deletion | . | KRT10 | exonic frameshift deletion |
| chr17 | 38978654 | 38978654 | A | G | SNP | . | KRT10 | exonic nonsynonymous SNV |
| chr17 | 40048483 | 40048535 | GCATAGTTCATGGTCCCCACCCCCCACCCTCCAGAGCCCAGTGATCTACCTGG | - | Deletion | . | ACLY | exonic frameshift deletion |
| chr17 | 76424616 | 76424616 | C | T | SNP | rs35597902 | DNAH17 | exonic nonsynonymous SNV |
| chr17 | 76482323 | 76482323 | C | T | SNP | rs377570773 | DNAH17 | exonic nonsynonymous SNV |
| chr17 | 76510860 | 76510860 | G | A | SNP | rs548985742 | DNAH17 | exonic nonsynonymous SNV |
| chr17 | 76510974 | 76510974 | G | A | SNP | rs78098467 | DNAH17 | exonic nonsynonymous SNV |
| chr18 | 12986927 | 12986929 | TCC | - | Deletion | rs769046151 | SEH1L | exonic nonframeshift deletion |
| chr18 | 12986964 | 12986964 | G | A | SNP | rs142773894 | SEH1L | exonic nonsynonymous SNV |
| chr18 | 44774764 | 44774764 | A | C | SNP | . | SKOR2 | exonic nonsynonymous SNV |
| chr19 | 1790763 | 1790763 | C | T | SNP | rs186722296 | ATP8B3 | exonic nonsynonymous SNV |
| chr19 | 1808227 | 1808229 | GAT | - | Deletion | rs141216755 | ATP8B3 | exonic nonframeshift deletion |
| chr19 | 55344239 | 55344239 | T | G | SNP | rs1130476 | KIR2DS4 | exonic nonsynonymous SNV |
| chr19 | 55346534 | 55346534 | G | C | SNP | rs112522228 | KIR2DS4 | exonic nonsynonymous SNV |
| chr19 | 55346565 | 55346565 | G | T | SNP | rs1130478 | KIR2DS4 | exonic nonsynonymous SNV |
| chr19 | 55358645 | 55358645 | C | T | SNP | rs144486451 | KIR2DS4 | exonic nonsynonymous SNV |
| chr19 | 55358654 | 55358654 | - | GC | Insertion | . | KIR2DS4 | exonic frameshift insertion |
| chr19 | 55358655 | 55358655 | A | G | SNP | rs113664264 | KIR2DS4 | exonic nonsynonymous SNV |
| chr19 | 55358658 | 55358659 | TG | - | Deletion | . | KIR2DS4 | exonic frameshift deletion |
| chr19 | 55358658 | 55358658 | T | C | SNP | rs72489166 | KIR2DS4 | exonic nonsynonymous SNV |
| chr19 | 55358686 | 55358686 | A | C | SNP | rs112697729 | KIR2DS4 | exonic nonsynonymous SNV |
| chr19 | 55358734 | 55358734 | G | A | SNP | . | KIR2DS4 | exonic nonsynonymous SNV |
| chr19 | 55358736 | 55358736 | - | A | Insertion | rs145114829 | KIR2DS4 | exonic frameshift insertion |
| chr19 | 55359211 | 55359211 | G | T | SNP | rs80015348 | KIR2DS4 | exonic nonsynonymous SNV |
| chr19 | 55359227 | 55359227 | A | G | SNP | rs78647568 | KIR2DS4 | exonic nonsynonymous SNV |
| chr19 | 55359244 | 55359245 | AC | - | Deletion | rs138992022 | KIR2DS4 | exonic frameshift deletion |
| chr2 | 61315638 | 61315638 | - | TA | Insertion | rs371063167 | KIAA1841 | exonic frameshift insertion |
| chr2 | 61361271 | 61361271 | G | T | SNP | rs150868433 | KIAA1841 | splicing |
| chr2 | 74642265 | 74642265 | - | CGCGGAGGGGCGGGTGGCGCCGCC | Insertion | rs768089535 | C2orf81 | exonic nonframeshift insertion |
| chr2 | 171570749 | 171570749 | A | C | SNP | rs74923148 | LOC440925 | exonic nonsynonymous SNV |
| chr2 | 172305177 | 172305177 | C | T | SNP | rs192861143 | DCAF17 | intronic |
| chr2 | 233346498 | 233346498 | C | T | SNP | rs142492002 | ECEL1 | exonic nonsynonymous SNV |
| chr20 | 3673194 | 3673194 | G | T | SNP | rs3746636 | SIGLEC1 | exonic nonsynonymous SNV |
| chr20 | 3673296 | 3673296 | A | G | SNP | rs200137244 | SIGLEC1 | exonic nonsynonymous SNV |
| chr20 | 3683874 | 3683874 | G | A | SNP | rs76254218 | SIGLEC1 | exonic nonsynonymous SNV |
| chr20 | 3687141 | 3687141 | C | A | SNP | rs150358287 | SIGLEC1 | exonic stopgain |
| chr21 | 47766840 | 47766840 | G | A | SNP | rs186701249 | PCNT | exonic nonsynonymous SNV |
| chr21 | 47786597 | 47786597 | T | C | SNP | rs199515373 | PCNT | exonic nonsynonymous SNV |
| chr21 | 47809204 | 47809204 | A | C | SNP | rs199787861 | PCNT | exonic nonsynonymous SNV |
| chr21 | 47819524 | 47819524 | G | T | SNP | rs775413533 | PCNT | exonic nonsynonymous SNV |
| chr21 | 47831454 | 47831454 | C | T | SNP | . | PCNT | exonic nonsynonymous SNV |
| chr21 | 47848307 | 47848307 | A | G | SNP | . | PCNT | splicing |
| chr21 | 47855894 | 47855896 | GAA | - | Deletion | rs562568796 | PCNT | exonic nonframeshift deletion |
| chr22 | 50315905 | 50315942 | AGTCAGGACCGGCCTCTCCGATTCTTACGCCCCTCAGC | - | Deletion | rs747744457 | CRELD2 | exonic frameshift deletion |
| chr22 | 50315933 | 50315933 | G | - | Deletion | rs759961951 | CRELD2 | exonic frameshift deletion |
| chr22 | 50315937 | 50315973 | CTCAGCAGTCAGGACCGGCCTCTCCGATTCTTACCCG | - | Deletion | rs758608747 | CRELD2 | exonic frameshift deletion |
| chr3 | 52027854 | 52027859 | CCTTGG | - | Deletion | rs368982942 | RPL29 | exonic nonframeshift deletion |
| chr3 | 52027859 | 52027859 | - | CCTTGG | Insertion | . | RPL29 | exonic nonframeshift insertion |
| chr3 | 132438619 | 132438619 | G | A | SNP | rs142663818 | NPHP3 | exonic nonsynonymous SNV |
| chr4 | 1388346 | 1388346 | - | CACACGTGCCCATGTGGAGTGCCCGCCTGCT | Insertion | rs773907017 | CRIPAK | exonic frameshift insertion |
| chr4 | 1388348 | 1388348 | - | AGTGCCCATGTGGAGTGCC | Insertion | rs751694408 | CRIPAK | exonic frameshift insertion |
| chr4 | 1388350 | 1388350 | - | CCTGCTCACACA | Insertion | . | CRIPAK | exonic nonframeshift insertion |
| chr4 | 1388350 | 1388350 | - | TGCCCATGTGGAGTGCCCGCCTGCTCACACA | Insertion | rs750778284 | CRIPAK | exonic frameshift insertion |
| chr4 | 38016343 | 38016343 | - | GCC | Insertion | . | TBC1D1 | exonic nonframeshift insertion |
| chr4 | 38016344 | 38016344 | - | CGC | Insertion | . | TBC1D1 | exonic nonframeshift insertion |
| chr4 | 38016346 | 38016346 | - | G | Insertion | . | TBC1D1 | exonic frameshift insertion |
| chr4 | 38016355 | 38016356 | AA | - | Deletion | . | TBC1D1 | exonic frameshift deletion |
| chr4 | 38138931 | 38138931 | C | T | SNP | rs575644987 | TBC1D1 | exonic nonsynonymous SNV |
| chr4 | 155156542 | 155156542 | G | A | SNP | rs61741046 | DCHS2 | exonic nonsynonymous SNV |
| chr4 | 155156803 | 155156803 | A | G | SNP | . | DCHS2 | exonic nonsynonymous SNV |
| chr4 | 155157828 | 155157828 | T | C | SNP | rs778584525 | DCHS2 | exonic nonsynonymous SNV |
| chr4 | 155237124 | 155237124 | G | C | SNP | . | DCHS2 | exonic nonsynonymous SNV |
| chr5 | 65083952 | 65083952 | A | C | SNP | rs201553806 | NLN | exonic nonsynonymous SNV |
| chr5 | 65088487 | 65088487 | - | T | Insertion | rs766824315 | NLN | exonic frameshift insertion |
| chr5 | 75699404 | 75699404 | C | A | SNP | rs569312379 | IQGAP2 | exonic nonsynonymous SNV |
| chr5 | 75906923 | 75906923 | C | A | SNP | rs3822530 | IQGAP2 | exonic nonsynonymous SNV |
| chr5 | 75991420 | 75991420 | C | T | SNP | rs17681908 | IQGAP2 | exonic nonsynonymous SNV |
| chr6 | 29857108 | 29857108 | C | G | SNP | rs754344993 | HLA-H | exonic nonsynonymous SNV |
| chr6 | 29857116 | 29857116 | G | A | SNP | rs201656279 | HLA-H | exonic nonsynonymous SNV |
| chr6 | 29857119 | 29857119 | A | G | SNP | rs200033654 | HLA-H | exonic nonsynonymous SNV |
| chr6 | 29976857 | 29976857 | G | C | SNP | rs9261114 | HLA-J | exonic nonsynonymous SNV |
| chr6 | 29976875 | 29976875 | G | A | SNP | . | HLA-J | exonic stopgain |
| chr6 | 30782029 | 30782029 | C | T | SNP | rs116942105 | AK098012 | exonic nonsynonymous SNV |
| chr6 | 30782245 | 30782245 | G | A | SNP | rs75666334 | AK098012 | exonic stopgain |
| chr6 | 30782325 | 30782325 | C | - | Deletion | rs560769326 | AK098012 | exonic frameshift deletion |
| chr6 | 30782328 | 30782328 | C | G | SNP | rs561184672 | AK098012 | exonic nonsynonymous SNV |
| chr6 | 31597380 | 31597380 | C | G | SNP | rs75044749 | PRRC2A | exonic nonsynonymous SNV |
| chr6 | 31601293 | 31601293 | G | A | SNP | rs768146814 | PRRC2A | exonic nonsynonymous SNV |
| chr6 | 31601380 | 31601380 | C | T | SNP | rs139066704 | PRRC2A | exonic nonsynonymous SNV |
| chr6 | 31603189 | 31603189 | G | A | SNP | rs11538264 | PRRC2A | exonic nonsynonymous SNV |
| chr6 | 31603453 | 31603453 | C | T | SNP | rs115028652 | PRRC2A | exonic nonsynonymous SNV |
| chr6 | 31604513 | 31604513 | C | A | SNP | rs114580964 | PRRC2A | exonic nonsynonymous SNV |
| chr6 | 31604894 | 31604894 | C | T | SNP | rs34137317 | PRRC2A | exonic nonsynonymous SNV |
| chr6 | 133118216 | 133118216 | A | G | SNP | rs41286192 | SLC18B1 | exonic nonsynonymous SNV |
| chr7 | 75048716 | 75048716 | G | T | SNP | rs377567968 | POM121C | exonic nonsynonymous SNV |
| chr7 | 75052129 | 75052129 | G | A | SNP | rs587770701 | POM121C | exonic nonsynonymous SNV |
| chr7 | 102182021 | 102182021 | C | A | SNP | rs774619252 | POLR2J3 | exonic nonsynonymous SNV |
| chr7 | 102182031 | 102182031 | G | A | SNP | rs4729825 | POLR2J3 | exonic nonsynonymous SNV |
| chr7 | 102212929 | 102212929 | C | G | SNP | rs779880850 | POLR2J3 | exonic nonsynonymous SNV |
| chr7 | 151841837 | 151841837 | T | G | SNP | . | KMT2C | exonic nonsynonymous SNV |
| chr7 | 151878800 | 151878800 | G | A | SNP | . | KMT2C | exonic nonsynonymous SNV |
| chr7 | 151879593 | 151879593 | - | TGC | Insertion | . | KMT2C | exonic nonframeshift insertion |
| chr7 | 151927023 | 151927023 | G | C | SNP | rs58528565 | KMT2C | exonic stopgain |
| chr7 | 151927067 | 151927067 | T | C | SNP | rs60244562 | KMT2C | exonic nonsynonymous SNV |
| chr7 | 151935799 | 151935799 | A | G | SNP | rs199839047 | KMT2C | exonic nonsynonymous SNV |
| chr7 | 151935866 | 151935866 | G | A | SNP | rs112515611 | KMT2C | exonic nonsynonymous SNV |
| chr7 | 151935871 | 151935871 | C | A | SNP | rs111493987 | KMT2C | exonic nonsynonymous SNV |
| chr7 | 151935910 | 151935910 | C | T | SNP | rs4024419 | KMT2C | exonic nonsynonymous SNV |
| chr7 | 151945225 | 151945225 | T | C | SNP | rs202098135 | KMT2C | exonic nonsynonymous SNV |
| chr7 | 151945228 | 151945228 | G | A | SNP | rs200184971 | KMT2C | exonic nonsynonymous SNV |
| chr7 | 151970951 | 151970951 | C | T | SNP | rs201009236 | KMT2C | exonic nonsynonymous SNV |
| chr8 | 12453641 | 12453641 | C | T | SNP | rs201507549 | LOC729732 | exonic nonsynonymous SNV |
| chr8 | 12516979 | 12516979 | G | C | SNP | rs187236124 | LOC729732 | exonic nonsynonymous SNV |
| chr9 | 40702839 | 40702839 | G | C | SNP | rs201863232 | SPATA31A3 | exonic nonsynonymous SNV |
| chr9 | 40702889 | 40702889 | T | G | SNP | . | SPATA31A3 | exonic nonsynonymous SNV |
| chr9 | 40706227 | 40706227 | G | A | SNP | rs759025747 | SPATA31A3 | exonic nonsynonymous SNV |
| chr9 | 69390009 | 69390009 | A | C | SNP | . | ANKRD20A4 | exonic nonsynonymous SNV |
| chr9 | 69420392 | 69420392 | C | T | SNP | rs201457457 | ANKRD20A4 | exonic stopgain |
| chr9 | 69423476 | 69423476 | A | G | SNP | rs373163693 | ANKRD20A4 | exonic nonsynonymous SNV |
| chr9 | 69423770 | 69423770 | C | T | SNP | rs200208176 | ANKRD20A4 | exonic nonsynonymous SNV |
| chr9 | 69423792 | 69423792 | A | T | SNP | rs75696372 | ANKRD20A4 | exonic nonsynonymous SNV |
| chr9 | 69423887 | 69423887 | A | G | SNP | rs199906260 | ANKRD20A4 | exonic nonsynonymous SNV |
| chr9 | 90534191 | 90534201 | CATCTTGTCTC | - | Deletion | rs758896527 | SPATA31C1 | exonic frameshift deletion |
| chr9 | 90534204 | 90534204 | - | GC | Insertion | rs747991568 | SPATA31C1 | exonic frameshift insertion |
| chr9 | 90534206 | 90534212 | TGTCCAA | - | Deletion | rs776971060 | SPATA31C1 | exonic frameshift deletion |
| chr9 | 90534206 | 90534206 | - | CATCTTGTCTCCCAGC | Insertion | . | SPATA31C1 | exonic frameshift insertion |
| chr9 | 90534212 | 90534212 | A | G | SNP | rs654472 | SPATA31C1 | exonic nonsynonymous SNV |
| chr9 | 90536994 | 90536994 | A | C | SNP | rs559309371 | SPATA31C1 | exonic nonsynonymous SNV |
| chr9 | 99961638 | 99961638 | C | G | SNP | rs41316516 | ZNF322 | exonic nonsynonymous SNV |
| chr9 | 139747739 | 139747739 | T | A | SNP | rs186097368 | MAMDC4 | exonic nonsynonymous SNV |
| chr9 | 139749495 | 139749495 | G | A | SNP | rs185932750 | MAMDC4 | exonic nonsynonymous SNV |
| chr9 | 139751739 | 139751743 | GCCCA | - | Deletion | . | MAMDC4 | exonic frameshift deletion |
| chr9 | 139753246 | 139753246 | C | T | SNP | rs116894068 | MAMDC4 | exonic nonsynonymous SNV |
| chrX | 103495090 | 103495090 | - | GCGCCATGGGCGGCCCGGGTGGCACAC | Insertion | . | ESX1 | exonic nonframeshift insertion |
| chrX | 103495090 | 103495090 | - | GCGCCATGGGCGGCCCGGGTGGCAGAGGCGCCATGGGCGGCCCGGGTGGCACAC | Insertion | . | ESX1 | exonic nonframeshift insertion |
| chrX | 103495161 | 103495161 | G | - | Deletion | . | ESX1 | exonic frameshift deletion |
| chrX | 103495165 | 103495217 | GGCACAGGCGCCATGGGCGGCCCGGTTGGCACAGGCGCCATGGGCGGCCAGGG | - | Deletion | . | ESX1 | exonic frameshift deletion |

**Supplementary Table 2. Pathogenic genes for DR.**

| **Symbol** | **Related to diabetic complication** | **Entrez Gene Name** | **Entrez Gene ID for Human** |
| --- | --- | --- | --- |
| C2orf81 | undetermined | chromosome 2 open reading frame 81 | 388963 |
| CRELD2 | determined | cysteine rich with EGF like domains 2 | 79174 |
| DCHS2 | undetermined | dachsous cadherin-related 2 | 54798 |
| DNAH17 | determined | dynein axonemal heavy chain 17 | 8632 |
| GPRC5A | determined | G protein-coupled receptor class C group 5 member A | 9052 |
| KIAA1841 | undetermined | KIAA1841 | 84542 |
| LOC729732 | undetermined | uncharacterized LOC729732 | 729732 |
| NLN | determined | neurolysin | 57486 |
| NPHP3 | determined | nephrocystin 3 | 27031 |
| PAPSS2 | determined | 3'-phosphoadenosine 5'-phosphosulfate synthase 2 | 9060 |
| POLR2J2/POLR2J3 | undetermined | RNA polymerase II subunit J2 | 246721\|548644 |
| PTPRF | determined | protein tyrosine phosphatase receptor type F | 5792 |
| TBC1D1 | determined | TBC1 domain family member 1 | 23216 |
| VPS13D | determined | vacuolar protein sorting 13 homolog D | 55187 |

**Supplementary Table 3. Pathogenic genes for DM-DR.**

| **Symbol** | **Related to diabetic complication** | **Entrez Gene Name** | **Entrez Gene ID for Human** |
| --- | --- | --- | --- |
| ATP8B3 | determined | ATPase phospholipid transporting 8B3 | 148229 |
| CUX2 | determined | cut like homeobox 2 | 23316 |
| ECEL1 | determined | endothelin converting enzyme like 1 | 9427 |
| EFCAB2 | undetermined | EF-hand calcium binding domain 2 | 84288 |
| HLA-H | undetermined | major histocompatibility complex, class I, H (pseudogene) | 3136 |
| HLA-J | determined | major histocompatibility complex, class I, J (pseudogene) | 3137 |
| KIR2DS2 (includes others) | determined | killer cell immunoglobulin like receptor, two Ig domains and short cytoplasmic tail 2 | 3806\|3809\|3810\|100132285 |
| POM121/POM121C | determined | POM121 transmembrane nucleoporin | 9883\|100101267 |
| SPATA31A6 (includes others) | undetermined | SPATA31 subfamily A member 6 | 26165\|389730\|647060\|727830\|727905 |

**Supplementary Table 4. Protective genes for DR and DM-DR.**

| **Symbol** | **Related to diabetic complication** | **Entrez Gene Name** | **Entrez Gene ID for Human** |
| --- | --- | --- | --- |
| ANKRD20A4P (includes others) | undetermined | ankyrin repeat domain 20 family member A3, pseudogene | 84210\|441425\|441430\|728747 |
| ESX1 | determined | ESX homeobox 1 | 80712 |
| GP1BA | determined | glycoprotein Ib platelet subunit alpha | 2811 |
| KMT2C | determined | lysine methyltransferase 2C | 58508 |
| MAMDC4 | determined | MAM domain containing 4 | 158056 |
| PCNT | determined | pericentrin | 5116 |
| PRAMEF1 (includes others) | determined | PRAME family member 1 | 65121\|65122\|400736 |
| PRRC2A | determined | proline rich coiled-coil 2A | 7916 |
| SPATA31C1 | undetermined | SPATA31 subfamily C member 1 | 441452 |
| LINC00243 | undetermined | long intergenic non-protein coding RNA 243 | 401247 |
